# Production of Instant Linden Tea (*Tilia* spp.) Powder: Integrating Compound‐Level Infusion Kinetics With Response Surface‐Optimized Spray Drying

**DOI:** 10.1002/fsn3.72018

**Published:** 2026-06-12

**Authors:** Evren Altiok, Ceyhun Kasapoglu

**Affiliations:** ^1^ Genetics and Bioengineering Department, Engineering Faculty Giresun University Giresun Türkiye; ^2^ Food Engineering Department, Engineering Faculty Istanbul Aydin University İstanbul Türkiye

**Keywords:** antioxidant activity, catechins, instant tea, linden tea, response surface methodology, spray drying

## Abstract

Producing instant herbal teas with consistent bioactive composition requires both efficient extraction and controlled drying to prevent compound degradation. This study developed instant linden tea (*Tilia* spp.) by integrating compound‐level infusion kinetics with response surface‐optimized spray drying. Monitoring individual polyphenols during hot‐water infusion revealed that extraction at 80°C for 45 min resulted in the highest recovery of major flavonoids and catechins while minimizing thermal epimerization. This optimized infusion was subsequently spray‐dried according to a Box–Behnken experimental design to evaluate the effects of inlet air temperature (150°C–170°C), feed rate (6.5–16.5 mL min^−1^), and maltodextrin concentration (10%–20%). Inlet temperature and maltodextrin concentration significantly influenced powder yield and antioxidant activity, whereas feed rate had no significant effect on these responses. Multi‐response optimization identified optimal conditions at 170°C inlet temperature, 10 mL min^−1^ feed rate, and 10% maltodextrin, yielding a powder with 40.33% yield, 3.97% moisture content, 34.39 mmol Trolox g^−1^ antioxidant activity, and 109 mg GAE g^−1^ total phenolics. HPLC analysis confirmed that the phenolic profile of the reconstituted powder closely matched that of the fresh infusion, with some compounds (catechin, kaempferol, EGCG) showing higher concentrations in the powder due to limited thermal degradation during rapid drying. The resulting powder exhibited excellent solubility (≈99.2%) and was well accepted in sensory evaluation. These findings suggest that integrating compound‐level infusion control with optimized spray drying can produce an instant linden tea powder with preserved bioactive composition and functional quality.

## Graphical abstract

Linden tea infusion kinetics were optimized at 80°C for 45 min to maximize flavonoid extraction. Optimal spray‐drying conditions (170°C, 10 mL/min, 10% maltodextrin) were determined using a Box‐Behnken design, yielding an instant powder with excellent solubility and preserved phenolic profile.

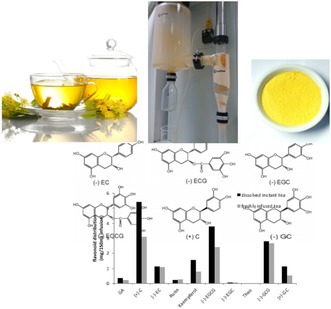

## 1 Introduction

Tea is a widely consumed beverage whose leaves contain phenolic compounds with potent antioxidant activity. Recent studies indicate that regular tea consumption is associated with a 10%–14% reduction in the risk of cardiovascular disease and type 2 diabetes, along with protective effects against certain cancers and age‐related degeneration (Yang et al. 2025; Kim and Je 2024; Cabrera et al. 2006; Du et al. 2012). However, these benefits are tied to a daily polyphenol intake roughly equivalent to 1.5–2 cups of tea, but the actual amount varies greatly. It depends on the type of tea, where it was grown, seasonal changes in the plant's composition, and even how it's brewed (Khan and Mukhtar 2018). Traditional brewing, while common, requires preparation time and does not guarantee a consistent dose of bioactive compounds from one cup to the next (Mazár et al. 2025; Çelik 2023). This is where instant tea powders offer an alternative. By providing a standardized product, they address the challenge of unpredictability, delivering not only convenience, but also a more reliable dose of bioactive compounds with each serving (Mazár et al. 2025; Thi Anh Dao et al. 2021). This health potential has shifted consumer expectations. People no longer drink tea solely for its taste; they also seek functional benefits (Huda et al. 2024). Instant tea powders dissolve instantly in hot water, occupy less space, remain stable on the shelf, and deliver a consistently standardized product (Thi Anh Dao et al. 2021; Çopur et al. 2019). One promising candidate for instant tea production is linden (*Tilia* spp.), known for its rich phenolic content and health benefits.

Linden has been valued for centuries in traditional medicine for its anti‐inflammatory, antioxidant, sedative, and anxiolytic properties. These benefits are now attributed to its rich phenolic profile (Ilyasoğlu and Arpa Zemzemoğlu 2023; Zhou et al. 2025). Its flowers and bracts are characterized by flavonol glycosides, particularly rutin and kaempferol derivatives, along with flavan‐3‐ols such as catechin and epicatechin, and procyanidins (Toker et al. 2001; Pavlović et al. 2020; Červenka et al. 2023). The transfer of phenolic compounds from dried linden inflorescences (flowers and bracts) into the infusion depends critically on brewing conditions. Temperature and duration influence not only the quantity of compounds extracted, but also their molecular stability and potential for transformation once in solution. Research has shown that while most commercial teas are steeped for only 3 min as package instructions suggest, this is far from sufficient to reach the full phenolic potential of linden. Optimal extraction of bioactive compounds typically requires higher temperatures (80°C–90°C) and longer infusion periods (İnanç and Yüksel 2018; Saklar et al. 2015). Furthermore, the phenolic profile is not static during infusion. Sensitive molecules like catechins can undergo reversible epimerization or thermal degradation, altering their chemical forms and, consequently, their bioactivity (Wang et al. 2000; Vuong et al. 2010). However, the extraction behavior of individual phenolic compounds from linden as a function of infusion time has not been systematically characterized. In particular, quantitative data on the thermal epimerization of catechins during prolonged infusion and how this affects the overall phenolic profile are not available for linden. Without this detail, designing extraction processes that preserve both the quantity and quality of bioactive compounds and developing standardized products remains a challenge.

Spray drying is the most widely used industrial technology for producing instant tea powders, valued for its rapid conversion of liquid extracts into dry form and its scalability (Thi Anh Dao et al. 2021; Mazár et al. 2025). Key process parameters (e.g., inlet air temperature, feed rate, and carrier agent concentration) critically influence both the physicochemical properties of the final product and the retention of bioactive compounds. Carrier agents such as maltodextrin play a particularly important role. They encapsulate sensitive phenolic compounds during high‐temperature drying, provide thermal protection, and reduce stickiness, thereby improving powder yield (Mazár et al. 2025; Nadali et al. 2022). The selected parameter ranges were based on preliminary experiments and literature findings. Inlet air temperatures (150°C–170°C) are commonly used for herbal tea drying to balance moisture removal and thermal degradation of phenolics (Ayaz et al. 2026; Tonon et al. 2011). Feed rates (6.5–16.5 mL/min) were chosen to evaluate droplet residence time effects on drying efficiency; similar ranges have been successfully applied to date and pineapple extracts (Sharma et al. 2023). Maltodextrin concentrations (10%–20% w/v) were selected because lower concentrations cause stickiness and low yield, while higher concentrations may mask the characteristic herbal flavor (Chong and Wong 2017). For linden tea, however, studies that integrate extraction kinetics with spray drying optimization remain scarce. While response surface methodology has been successfully applied to other herbal teas, such as green tea (Thi Anh Dao et al. 2021), no such integrated approach has been reported for linden. More importantly, what is missing is a compound‐level assessment of how individual flavonoids behave during drying and whether the reconstituted powder truly reflects the phenolic profile of the original infusion. Addressing this gap is essential for developing a standardized instant linden tea product with predictable bioactive composition.

Against this background, the present study aimed to develop a high‐quality instant linden tea powder by integrating compound‐level infusion kinetics with response surface‐optimized spray drying. In the first stage, the extraction behavior of individual phenolic compounds during hot‐water infusion will be monitored using HPLC to identify brewing conditions that maximize flavonoid recovery while limiting the release of bitter components such as theobromine. In the second stage, a Box–Behnken experimental design will be employed to optimize the effects of inlet air temperature, feed rate, and maltodextrin concentration on powder quality attributes. The novelty of this approach lies in its integration of compound‐level kinetic control with optimized drying, thereby moving beyond bulk phenolic measurements to track the fate of individual bioactive compounds throughout the process. The resulting powder will be evaluated in terms of yield, moisture content, antioxidant activity, HPLC‐based phenolic profile (compared with the fresh infusion), solubility, and sensory acceptability. Together, these analyses aim to provide a scientific basis for the industrial production of a standardized, functional, and sensorially acceptable instant linden tea.

## 2 Materials and Methods

### 2.1 Materials

Naturally dried linden inflorescences (flowers with bracts) and various commercial bagged herbal teas, including bagged linden tea, were obtained from a local market in Istanbul. The linden material was commercially packaged with a ministry‐approved production license, and its moisture content was determined as 10.46%.

HPLC standards, including (+)‐gallocatechin (GC), (+)‐catechin (C), (−)‐epicatechin (EC), (−)‐epigallocatechin (EGC), (−)‐epigallocatechin gallate (EGCG), (−)‐epicatechin gallate (ECG), (−)‐gallocatechin gallate (GCG), gallic acid (GA), theobromine, rutin, kaempferol, and caffeine, were acquired from Sigma‐Aldrich (Steinheim, Germany). Reagents used for antioxidant and total phenolic analysis, including ABTS (2,2′‐Azino‐bis(3‐Ethylbenzthiazoline‐6‐Sulfonic Acid)), Trolox ((±)‐6‐Hydroxy‐2,5,7,8‐tetramethylchromane‐2‐carboxylic acid), Folin–Ciocalteu reagent, potassium persulfate, and sodium carbonate, were also purchased from Sigma‐Aldrich. HPLC grade acetonitrile and acetic acid were obtained from Merck (Darmstadt, Germany). Food‐grade maltodextrin (20 dextrose equivalent) was obtained from Smart Kimya Ltd., Turkey, and used as the carrier agent during spray drying. Ultrapure water used in all experiments was prepared with Sartorius Ariumpro ultrapure water systems.

### 2.2 Infusion and Sample Preparation

#### 2.2.1 Optimization of Infusion Time: Kinetic and Comparative Analysis

Infusion kinetic studies were conducted to monitor the effect of infusion duration on the concentration and composition of individual phenolic compounds in linden tea infusion, antioxidant activity, and color of linden infusion. For each experiment, 2.00 (±0.02) g of the plant material was infused in 150 mL of water maintained at 80°C in a covered beaker to minimize evaporative loss, following the conventional hot water infusion procedure for herbal teas (Horžić et al. 2009). To obtain consistent extraction kinetic data, samples were collected at 2, 10, 30, 45, 60, 90, 120, and 150 min from independently prepared experimental setups, thereby maintaining a constant solid–liquid ratio. Immediately after sampling, each sample was rapidly cooled to 25°C ± 1°C to prevent thermal decomposition, centrifuged at 4000 rpm for 3 min to remove suspended solids, and then filtered through a 0.45 μm PTFE syringe filter. The resulting filtrates were subsequently analyzed via high‐performance liquid chromatography (HPLC) to quantify the temporal evolution of individual polyphenolic constituents. In parallel, the same filtrates were also assessed for their antioxidant capacity using the ABTS radical cation assay and for color parameters (e.g., L*, a*, b*) using a Lovibond tintometer, providing a comprehensive profile of the infusion's physicochemical changes over time (for detailed ABTS and color measurement procedures, see Section 2.3).

For comparison with commercially available products, the antioxidant activity of the studied linden tea infusion was evaluated alongside 10 other herbal tea infusions prepared under typical domestic brewing conditions. This comparison established a baseline for the antioxidant yield obtained from a standard short‐term infusion, which was subsequently used to quantify the enhancement achievable through optimized, longer extraction times in the kinetic study. All infusions were prepared by steeping a single tea bag in 150 mL of water at 80°C for 3 min, following the manufacturers' instructions, and their antioxidant activity was measured using the ABTS method.

#### 2.2.2 Preparation of Feed Solution for Spray Drying

The spray drying feed was prepared by infusing linden at a solid/liquid ratio of 80 g in 6 L of distilled water at 80°C for the optimized time determined in the present study. The extract was filtered and then concentrated under vacuum using a rotary evaporator (Heidolph Instruments, Germany) to increase soluble solids to a level suitable for spray drying, as reported for green tea extract processing (Susantikarn and Donlao 2016; Vuong et al. 2013). The vacuum pressure was gradually reduced from 150 mbar to 50 mbar to accelerate evaporation, and the extract was fed into the system in fed‐batch mode. The process was carried out at 40°C until a soluble solids content of 4.5° Brix was reached, with a total evaporation time of approximately 40 min. This concentration step was performed primarily to reduce extract volume and concentrate phenolic compounds. The concentrated extract was cooled to 25°C ± 1°C, filtered, and used as the feed solution for subsequent spray drying experiments.

### 2.3 Analytical Procedures

#### 2.3.1 High‐Performance Liquid Chromatography (HPLC) Analysis

Phenolic compounds in the infusions were characterized and quantified using an Agilent 1100 series HPLC system (Agilent Technologies, USA) equipped with a Lichrosphere RP C18 analytical column (250 mm × 4.0 mm; 5 μm particle size). The chromatographic conditions and gradient program were based on our previously established method (Altiok 2010). Detection was performed using a diode array detector (DAD). Chromatograms were recorded at 280 nm for flavan‐3‐ols (gallic acid, (+)‐catechin, (−)‐epicatechin, (−)‐epigallocatechin, (−)‐epicatechin gallate, (−)‐epigallocatechin gallate), at 256 nm for rutin, and at 368 nm for kaempferol. Chromatographic separation was achieved using a mobile phase consisting of (A) 5.5% acetic acid in water and (B) acetonitrile containing 20% mobile phase A. The gradient elution program at a flow rate of 1.0 mL/min was as follows: 16.5% B (0 min), 18% B (13 min), 25% B (25 min), 31.5% B (27 min), and 100% B (30 min), which was followed by an isocratic hold for 10 min. Calibration curves were prepared using external standards at multiple concentration levels. Quantification of individual catechins and flavonoids was performed using compound‐specific response factors (RF) and relative response factors (RRF). The RF and RRF values determined in this study, along with literature‐reported values used for comparison, are summarized in Tables S6 and S7.

#### 2.3.2 Total Phenolic Content (TPC) Determination

Total phenolic content was determined using the Folin–Ciocalteu method (Singleton et al. 1999). A stock solution of gallic acid (0.5 mg L^−1^) was prepared in distilled water and working standards were prepared at concentrations of 0.02, 0.03, 0.04, 0.05, and 0.06 mg mL^−1^ to construct the calibration curve. Briefly, 500 μL of diluted sample was mixed with 2.5 mL of Folin–Ciocalteu reagent (1:10 dilution) and 2.0 mL of 7.5% sodium carbonate solution. The mixture was incubated in the dark for 40 min at room temperature (25°C ± 1°C), and absorbance was measured at 725 nm using a UV–Vis spectrophotometer. Total phenolic content was calculated using the calibration curve equation (absorbance vs. gallic acid concentration) and expressed as mg gallic acid equivalents per gram of dry matter (mg GAE g^−1^).

#### 2.3.3 Total Antioxidant Activity (TEAC)

Antioxidant activity was determined using the ABTS radical cation decolorization assay (Re et al. 1999). The ABTS^+^ radical solution was prepared by reacting 7 mM ABTS with 2.45 mM potassium persulfate in distilled water and allowing the mixture to stand in the dark at room temperature for 16 h. The ABTS^+^ solution was then diluted with ethanol to an absorbance of 0.70 ± 0.02 at 734 nm. A Trolox stock solution (1 mM) was prepared in ethanol, and working standards were prepared at concentrations of 0, 50, 100, 150, 200, 250, and 500 μM to construct the calibration curve. Briefly, 30 μL of appropriately diluted sample or standard was mixed with 3 mL of diluted ABTS^+^ solution. After 6 min of incubation at 25°C ± 1°C, the absorbance reduction was measured at 734 nm using a UV–Vis spectrophotometer. Antioxidant activity was calculated using the calibration curve equation (absorbance reduction vs. Trolox concentration) and expressed as mmol Trolox equivalents per gram of sample (mmol Trolox g^−1^).

#### 2.3.4 Color Measurement

Color parameters (L*, a*, b*) were measured using a Lovibond PFXi‐950 tintometer (Lovibond Ltd., UK) based on the CIELAB color space. The L* value represents lightness, ranging from 0 (black) to 100 (white). The a* value indicates the position between green and red, where negative values correspond to green and positive values correspond to red. The b* value represents the position between blue and yellow, with negative values indicating blue and positive values indicating yellow (CIE, International Commission on Illumination 2004). Measurements were performed on filtered infusion samples collected at each time point during kinetic experiments. This enabled evaluation of color changes in relation to phenolic extraction and antioxidant activity.

#### 2.3.5 Product Characterization

Powder solubility was determined using a gravimetric method (ISO 2021). Briefly, 0.5 g of the sample was dissolved in 20 mL of distilled water, vortexed for 2 min, and centrifuged at 5000 rpm for 10 min. The supernatant was filtered and transferred to a pre‐weighed petri dish, dried in an oven at 105°C until constant weight. Solubility was calculated as the percentage of dissolved solids relative to the initial sample weight using the following equation:

$$\text{Solubility}\;\left(\%\right)=\left(\text{Weight of dried solids}/\text{Initial sample weight}\right)\times 100$$

To compare phenolic profiles, both spray‐dried powder and freshly prepared infusion were analyzed. Powder samples were dissolved in water, filtered, and diluted appropriately. Fresh infusion was prepared under optimized conditions and filtered before analysis. Phenolic composition was determined using the HPLC method described in Section 2.3.1. All measurements were performed in triplicate.

#### 2.3.6 Sensory Evaluation

Sensory evaluation was conducted using a trained panel of 10 assessors (5 male, 5 female, aged 25–45 years; ISO 2023), all of whom were frequent linden tea consumers familiar with its sensory characteristics and accustomed to selecting premium products. Four samples were evaluated: commercial bagged tea (3 min infusion), loose leaf tea (40 min infusion), and instant powder samples reconstituted at two concentrations (2 g/50 mL and 4 g/50 mL). The instant powders were produced by spray drying the 45‐min optimized infusion with 10% maltodextrin.

Samples (50 mL) were served at 60°C ± 2°C in coded cups with randomized presentation. Panelists evaluated appearance, aroma, taste, mouthfeel, aftertaste, and overall acceptability using a 9‐point hedonic scale (1 = extremely low/poor, 9 = extremely high/excellent). Unsalted crackers and water were provided for palate cleansing between samples.

### 2.4 Spray Drying Optimization and Statistical Analysis

Spray drying was performed using a laboratory‐scale spray dryer (Armfield, UK) equipped with a 0.5 mm nozzle atomizer. Atomization air pressure (3.5 bar) and airflow rate (22 m^3^ h^−1^) were kept constant throughout the experiments.

A three‐factor, three‐level Box–Behnken design was employed to optimize the spray drying conditions. The independent variables were inlet air temperature (150°C, 160°C, and 170°C), feed rate (6.5, 11.5, and 16.5 mL min^−1^), and maltodextrin concentration (10%, 15%, and 20%, w/v). The responses evaluated were powder yield (maximized), moisture content (minimized), antioxidant activity (maximized), and total phenolic content (maximized). Experimental design, model development, and multi‐response optimization using the desirability function approach were performed with Minitab (Version 17, Minitab Inc., USA). Second‐order polynomial models were fitted to the data, and model adequacy was assessed by analysis of variance, including regression coefficients, lack‐of‐fit tests (*p* > 0.05 indicating no significant lack of fit), *R* ^2^, and adjusted *R* ^2^. Three‐dimensional response surface and contour plots were generated using Python (Matplotlib) to visualize the effects of process variables and their interactions.

The validity of the response surface models was confirmed by conducting confirmatory experiments in triplicate at the predicted optimal conditions (inlet air temperature: 170°C, feed rate: 10 mL min^−1^, maltodextrin: 10%). The experimental mean values were compared with the predicted values, and the prediction accuracy was evaluated using the coefficient of variation, calculated as CV (%) = [(predicted value − experimental mean) / predicted value] × 100. Lower CV values indicate better agreement between predicted and experimental results, confirming the reliability of the optimization model. The repeatability of the confirmatory runs was additionally assessed by calculating the relative standard deviation (RSD = standard deviation/mean × 100) among the three replicates.

All experiments were performed in triplicate, and data are expressed as mean ± standard deviation. Prior to one‐way ANOVA, normality was checked using the Shapiro–Wilk test (*p* > 0.05) and homogeneity of variances was verified using Levene's test (*p* > 0.05). Statistical analyses were performed separately according to the experimental design. For the infusion kinetic study, comparison of commercial herbal teas, HPLC analysis of redissolved powders, and sensory evaluation, one‐way ANOVA followed by Tukey's honestly significant difference (HSD) test was applied at a 95% confidence level (*p* < 0.05) using Minitab. For the spray drying optimization, response surface models were developed based on the Box–Behnken design as described above. No preliminary RSM experiments were conducted; all Box–Behnken runs were performed as designed. Sensory data were additionally analyzed using multivariate analysis of variance (MANOVA) and principal component analysis (PCA) with Python.

## 3 Results and Discussion

### 3.1 Flavonoid Extraction and Infusion Kinetics

A kinetic evaluation was performed to determine the optimal infusion duration for *Tilia* spp. based on the extraction behavior of flavonoids and related phenolic compounds. These constituents are directly associated with key quality attributes, including color, clarity, sensory perception, and antioxidant capacity. The primary objective was to determine the optimal brewing time that maximizes flavonoid content while maintaining acceptable sensory qualities. The infusion temperature was maintained at a fixed value of 80°C to simulate conventional preparation conditions while ensuring efficient extraction of native phenolic compounds. This specific temperature has been previously reported to promote effective phenolic release while limiting excessive thermal degradation and undesired chemical alterations, such as catechin epimerization, which are more pronounced at boiling temperatures (Horžić et al. 2009; Khokhar and Magnusdottir 2002; Wang and Helliwell 2000). By keeping the temperature constant, the focus was placed on the kinetic behavior of the target compounds over time.

The extraction profiles indicated a progressive increase in phenolic compound concentrations during the initial infusion period, followed by compound‐specific transformations with extended exposure. Based on the overall flavonoid yield and stability considerations, an infusion time of 45 min was identified as optimal. The optimality of the 45‐min infusion time was established based on kinetic monitoring of flavonoid release at 80°C. This duration was selected because it provided the maximum extraction yield before reaching a concentration plateau. Beyond 45 min, no further significant increase in phenolic content was observed, while the potential for thermal degradation of sensitive compounds increased. At this time point, maximal extraction efficiency was achieved without significant degradation or structural alteration of major bioactive constituents. Comparable findings were reported for *Tilia cordata* , where equilibrium concentrations of phenolic compounds were reached within 40–60 min depending on temperature and diffusion conditions (İnanç and Yüksel 2018). The extraction kinetics of phenolic compounds from linden inflorescences followed Fick's first‐order diffusion model, as evidenced by the rapid initial increase in concentration followed by a gradual plateau. This diffusion‐controlled behavior is consistent with previously reported extraction profiles for herbal teas (Horžić et al. 2009).

**FIGURE 1:**
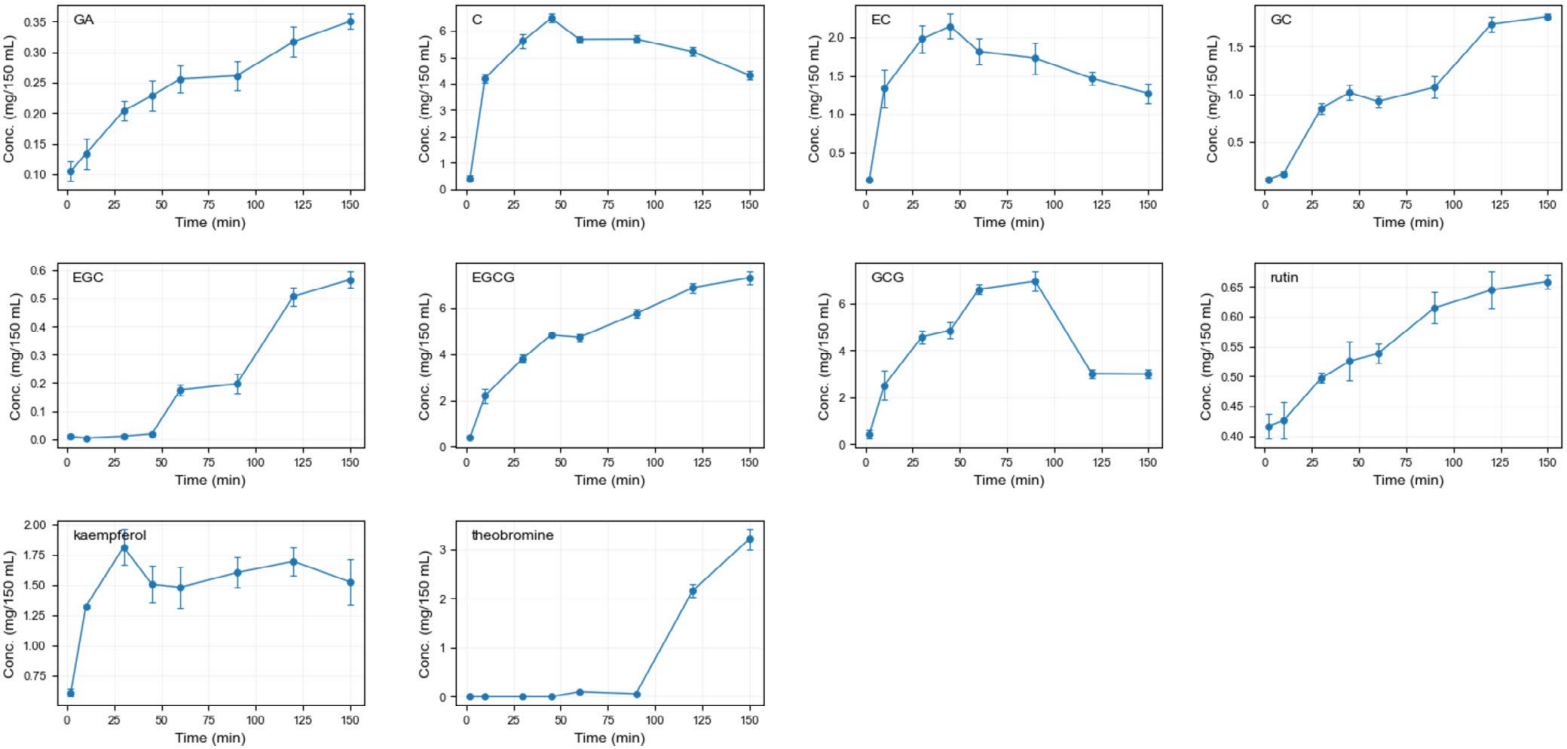
Extraction kinetics of individual phenolic compounds during linden tea infusion at 80°C. Each panel shows the concentration (mean ± SD, *n* = 3) of a specific compound over infusion time (2–150 min). C, catechin; EC, epicatechin; EGC, epigallocatechin; EGCG, epigallocatechin gallate; GA, gallic acid; GC, gallocatechin; GCG, gallocatechin gallate.

HPLC analysis confirmed the presence of gallic acid, (+)‐catechin, (−)‐epicatechin, rutin, and kaempferol in the infusion, consistent with previously reported phytochemical profiles of linden species (Červenka et al. 2023; Pavlović et al. 2020). As illustrated in Figure 1, the concentration of (+)‐catechin increased from 0.4 mg/150 mL at 2 min to 6.2 mg/150 mL at 45 min, while (−)‐epicatechin increased from 0.1 mg/150 mL to 2.1 mg/150 mL over the same period. However, prolonged infusion resulted in a significant decline in these compounds, with reductions of approximately 20% and 45%, respectively. This decrease coincided with a gradual increase in epigallocatechin concentration, suggesting thermally induced epimerization. These observations are consistent with previous reports describing the thermal instability of catechin epimers under elevated temperature conditions (Saklar et al. 2015; Wang et al. 2000). Vuong et al. (2010) further demonstrated that the epi‐structure of catechins becomes unstable and tends to epimerize at temperatures above 80°C. This epimerization reaction involves the conversion of epi‐forms (e.g., EGCG, ECG) to their corresponding non‐epi‐forms (e.g., GCG, CG) through a reversible temperature‐dependent mechanism (Wang et al. 2008). At 80°C, prolonged exposure provides sufficient activation energy for this transformation, explaining the gradual accumulation of epimerized products observed after 45 min of infusion. Although the infusion temperature in the present study was maintained at 80°C, extended exposure appears sufficient to promote epimerization reactions, resulting in a shift in the phenolic profile that reflects the dynamic balance between extraction, degradation, and structural conversion processes.

Further analysis revealed distinct compound‐specific trends. Epigallocatechin (EGC) increased sharply after 90 min (0.2 to 0.55 mg/150 mL), while gallocatechin gallate (GCG) peaked then declined sharply (from approximately 7 to 3 mg/150 mL). These changes indicate ongoing interconversion and degradation reactions that alter the phenolic profile over time. Specifically, gallated catechins like EGCG (which continued accumulating) and tri‐hydroxylated forms like EGC exhibit superior radical scavenging capacity due to their gallate moiety and higher hydroxyl group density (Łuczaj and Skrzydlewska 2005; Ze Xu et al. 2004). Therefore, infusion duration influences not only the total phenolic concentration but also the functional antioxidant potential of the resulting beverage.

In addition to polyphenolic compounds, the methylxanthine derivative theobromine was detected after prolonged infusion periods. Its increasing concentration was associated with observable changes in infusion clarity and foam formation, which may influence sensory perception and bitterness (Červenka et al. 2023). In contrast, caffeine was not detected in the analyzed samples, which is consistent with previous studies reporting that herbal infusions such as linden are characterized by very low or non‐detectable caffeine levels and may vary depending on plant origin and extraction conditions (Horžić et al. 2009).

### 3.2 Antioxidant Activity Kinetics and Commercial Comparison

**FIGURE 2:**
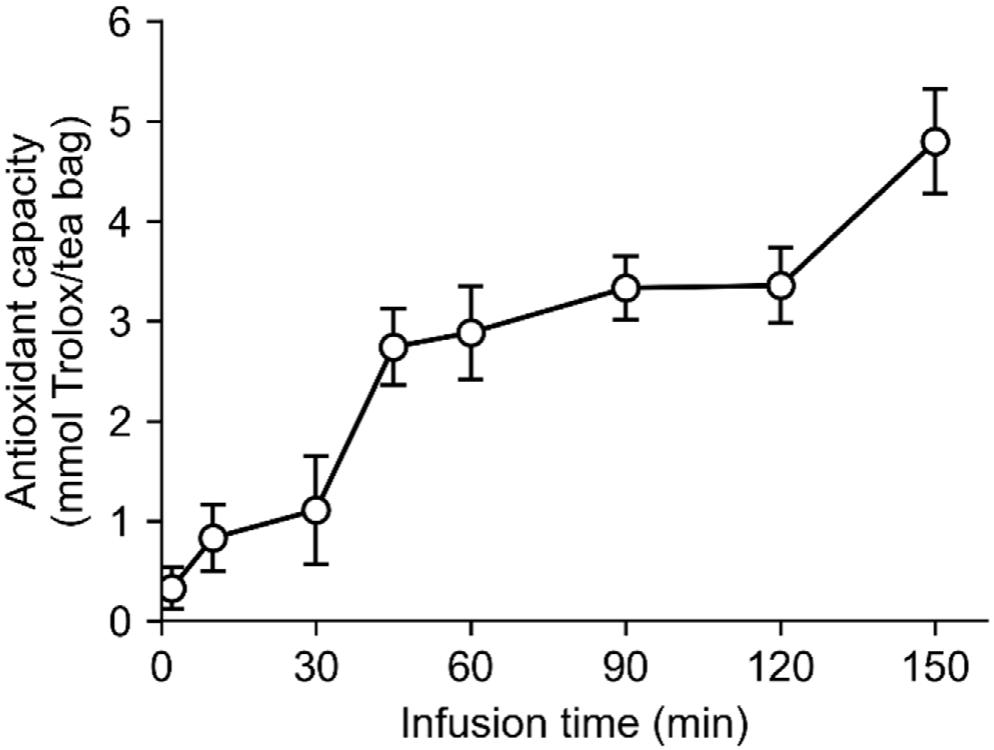
Antioxidant activity (TEAC) changes of linden infusion as a function of brewing time at 80°C. Values are expressed as mean ± SD.

The antioxidant capacity (TEAC) of *Tilia* spp. infusion increased with infusion time (Figure 2). During the first 45 min, the TEAC value rose nearly tenfold to reach 2.75 mmol Trolox/tea bag, corresponding to the period of maximal polyphenol release identified in the kinetic analysis (Section 3.1). This rapid increase reflects the efficient diffusion and solubilization of readily extractable phenolic compounds into the aqueous phase. Despite the observed decline in the concentrations of (+)‐catechin and (−)‐epicatechin beyond 45 min, the overall antioxidant capacity continued to increase, reaching a TEAC value of 4.5 mmol Trolox/tea bag at 150 min (Figure 2). This observation indicates that antioxidant capacity is governed not only by the concentration of individual compounds but also by the evolving composition and reactivity of the phenolic matrix. Continued extraction of other antioxidant constituents, including gallic acid, rutin, and kaempferol, contributed to the sustained increase in TEAC. In addition, structural transformations occurring during prolonged infusion, such as epimerization and conversion of catechins into alternative forms, likely generated compounds with comparable or enhanced radical scavenging activity. These findings demonstrate that infusion time influences both the quantitative and qualitative characteristics of the phenolic fraction, resulting in a progressive increase in antioxidant functionality even after peak concentrations of certain native catechins have been reached. These findings are consistent with previous reports on herbal infusions, where total antioxidant activity was strongly correlated with the overall phenolic profile rather than with any single compound (Atoui et al. 2005; Samaniego‐Sánchez et al. 2011).

Although a Pearson correlation analysis between individual phenolic compounds and TEAC values was not performed in this study, the temporal trends observed in Figures 1 and 2 suggest a strong association between flavonoid extraction and antioxidant capacity. The continued increase in TEAC despite the decline in certain catechins further indicates that other phenolic constituents, as well as epimerization products, contribute to the overall radical scavenging capacity. This interpretation is supported by previous studies showing that the antioxidant activity of herbal infusions is a function of the combined effect of multiple phenolic compounds, including flavonols, gallic acid derivatives, and epimerized catechins (Atoui et al. 2005; Łuczaj and Skrzydlewska 2005).

**FIGURE 3:**
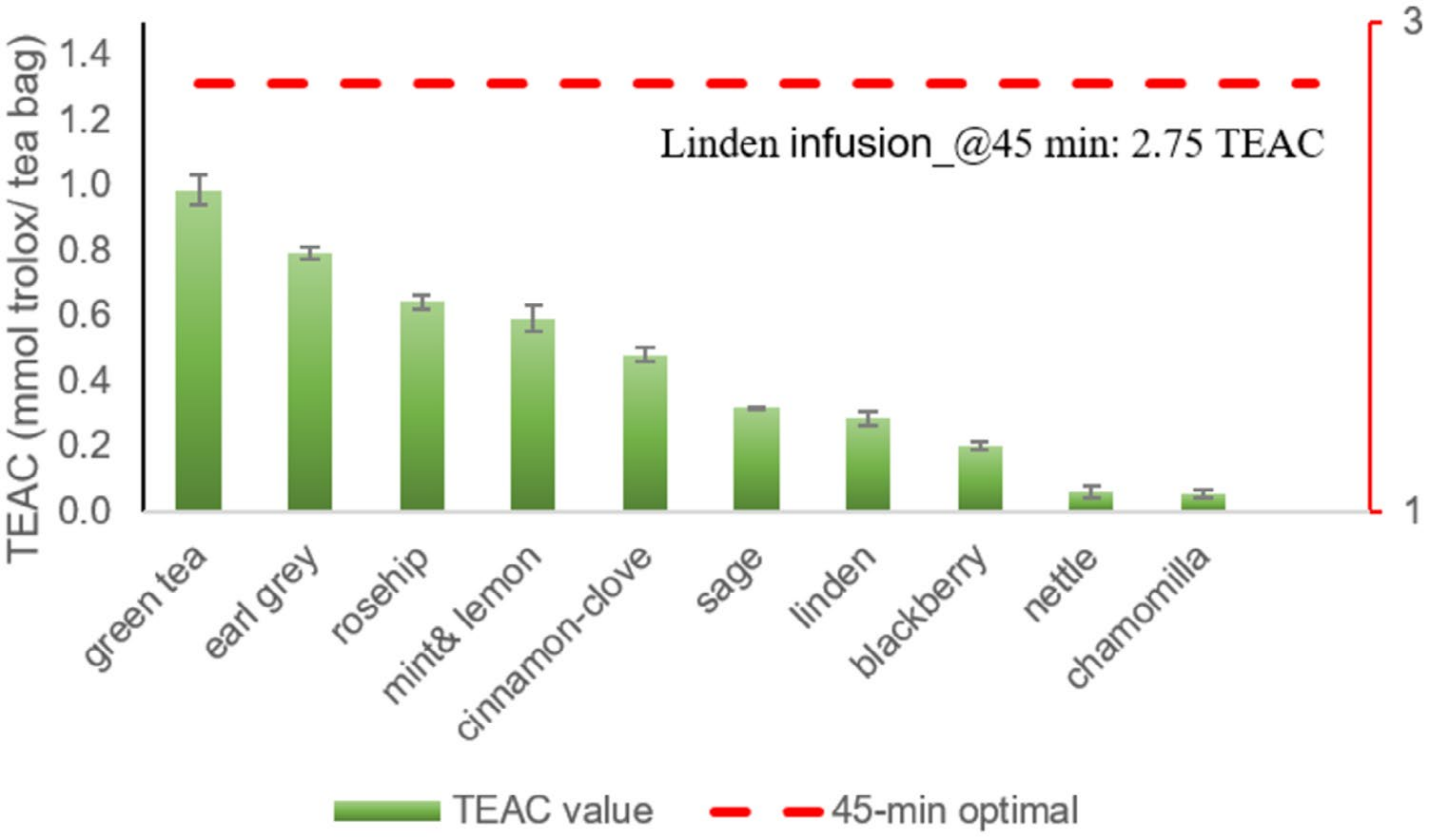
Antioxidant capacity of commercial bagged teas under different infusion protocol. TEAC values of commercial bagged teas after standard 3‐min hot water infusion (90°C). The red dashed line indicates the antioxidant capacity achieved with optimal 45‐min infusion of linden tea (2.75 mmol Trolox/tea bag), demonstrating significantly higher extraction efficiency with prolonged infusion time.

The importance of infusion duration became particularly evident when comparing antioxidant capacity under conventional and optimized preparation conditions (Figure 3). Infusion of linden tea for 3 min, consistent with commercial tea bag instructions, resulted in a TEAC value of 0.29 mmol Trolox/tea bag. In contrast, the optimized infusion time of 45 min produced a TEAC value of 2.75 mmol Trolox/tea bag, representing an approximately 9.5‐fold increase. This substantial difference confirms that short infusion periods do not allow sufficient time for the complete extraction of bioactive phenolic compounds. This time‐dependent enhancement is consistent with previous reports on tea infusions. For example, green tea infused at 80°C for 5 min exhibited a TEAC value of approximately 1.17 (Samaniego‐Sánchez et al. 2011), which remains considerably lower than the antioxidant capacity achieved under the optimized conditions in the present study. Furthermore, the optimized linden infusion exhibited between 2.8‐ and 50‐fold higher antioxidant capacity compared with various commercial bagged teas prepared under standard conditions, including green tea (TEAC = 0.98 mmol Trolox/tea bag) and Earl Gray tea (TEAC = 0.79 mmol Trolox/tea bag).

These results highlight infusion time as a key process variable controlling antioxidant extraction efficiency. Extended infusion promotes greater recovery of phenolic compounds and enables compositional shifts that enhance overall radical scavenging capacity. From a process optimization perspective, the identification of an appropriate infusion duration represents a critical step for maximizing the functional quality of linden tea extracts prior to further processing, such as spray drying.

### 3.3 Color Evolution During Infusion: Relationship With Chemical Composition and Sensory Quality

**FIGURE 4:**
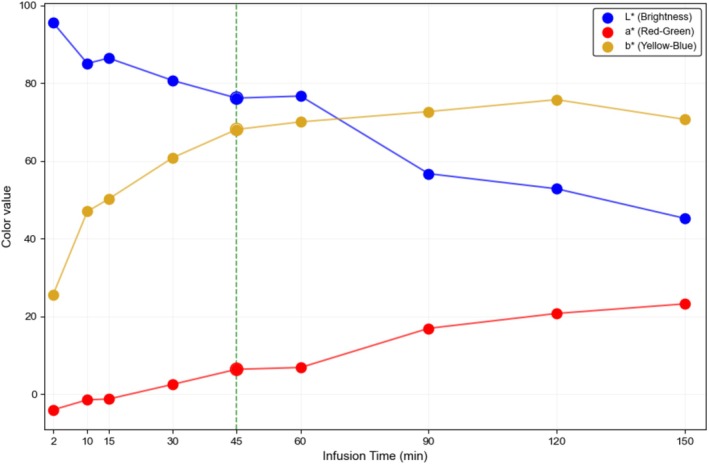
Changes in CIE L*a*b* color parameters during linden tea infusion. L* represents brightness (higher values indicate lighter color), a* indicates red‐green balance (positive values shift toward red), and b* shows yellow‐blue balance (positive values indicate yellow tones). The vertical dashed line marks the 45‐min infusion time, identified as optimal for balancing color development with phenolic extraction efficiency.

The visual appearance of herbal infusions is a critical quality attribute that reflects their chemical composition and influences consumer perception. To evaluate the effect of infusion time on visual quality, CIELAB color parameters (L*, a*, b*) were monitored throughout the extraction process (Figure 4). Lightness (L*) decreased progressively with infusion time, indicating gradual darkening of the infusion as phenolic compounds and other soluble constituents accumulated in the aqueous phase. This behavior is consistent with previous studies reporting that increased extraction and subsequent oxidation of polyphenolic compounds lead to higher light absorption and darker infusion color (Horžić et al. 2009; Wang et al. 2000). This darkening effect was moderate up to 45–60 min but became more pronounced at longer infusion times (90–120 min). The reduction in L* can be attributed primarily to increased concentrations of extracted polyphenols and the formation of more complex phenolic structures through oxidation, epimerization, and intermolecular interactions. These transformations promote the formation of higher molecular weight compounds with greater light absorption, resulting in reduced brightness and increased visual density of the infusion (Łuczaj and Skrzydlewska 2005).

The red–green coordinate (a*) shifted from negative to positive values between 30 and 45 min, indicating a transition from greenish to reddish tones, and continued to increase gradually thereafter. This color change coincides with the epimerization of catechins and changes in phenolic compound concentrations discussed in Section 3.1, suggesting a relationship between the infusion's chemical composition and its color development. Consistent with this, thermal epimerization and structural modification of catechins have been shown to significantly influence the optical properties of tea infusions (Saklar et al. 2015; Wang et al. 2008). The yellow–blue coordinate (b*) increased rapidly during the first 45 min and then plateaued, indicating that most yellow‐colored flavonoids are extracted within this period. This stabilization suggests that extraction of these compounds approaches equilibrium after 45 min, with limited further contribution to yellowness at longer infusion times. Similar trends have been reported in tea systems, where flavonoid extraction occurs predominantly during the early stages of infusion (Yadav et al. 2017; Samaniego‐Sánchez et al. 2011).

Taken together, these results indicate that approximately 45 min represents a colorimetric equilibrium point, at which the infusion achieves a balanced visual profile characterized by sufficient color intensity while maintaining acceptable brightness (indicated by the vertical line in Figure 4). Beyond this point, continued infusion results primarily in further darkening rather than additional improvement in color attributes. This darkening likely reflects ongoing compositional changes, including the transformation of phenolic compounds discussed in Section 3.1, which may affect visual clarity and perceived quality. The observed equilibrium behavior is consistent with kinetic studies of phenolic extraction in tea systems, where mass transfer approaches a plateau after extended infusion times (İnanç and Yüksel 2018).

These findings support the selection of 45 min as the optimal infusion time, not only from a compositional and antioxidant perspective (Sections 3.1 and 3.2) but also in terms of preserving a balanced and sensorially acceptable color profile. The convergence of chemical, functional, and colorimetric indicators at this time point reinforces its suitability as the optimal extraction condition for producing linden tea infusions.

### 3.4 Spray Drying Optimization and Product Quality Evaluation

**FIGURE 5:**
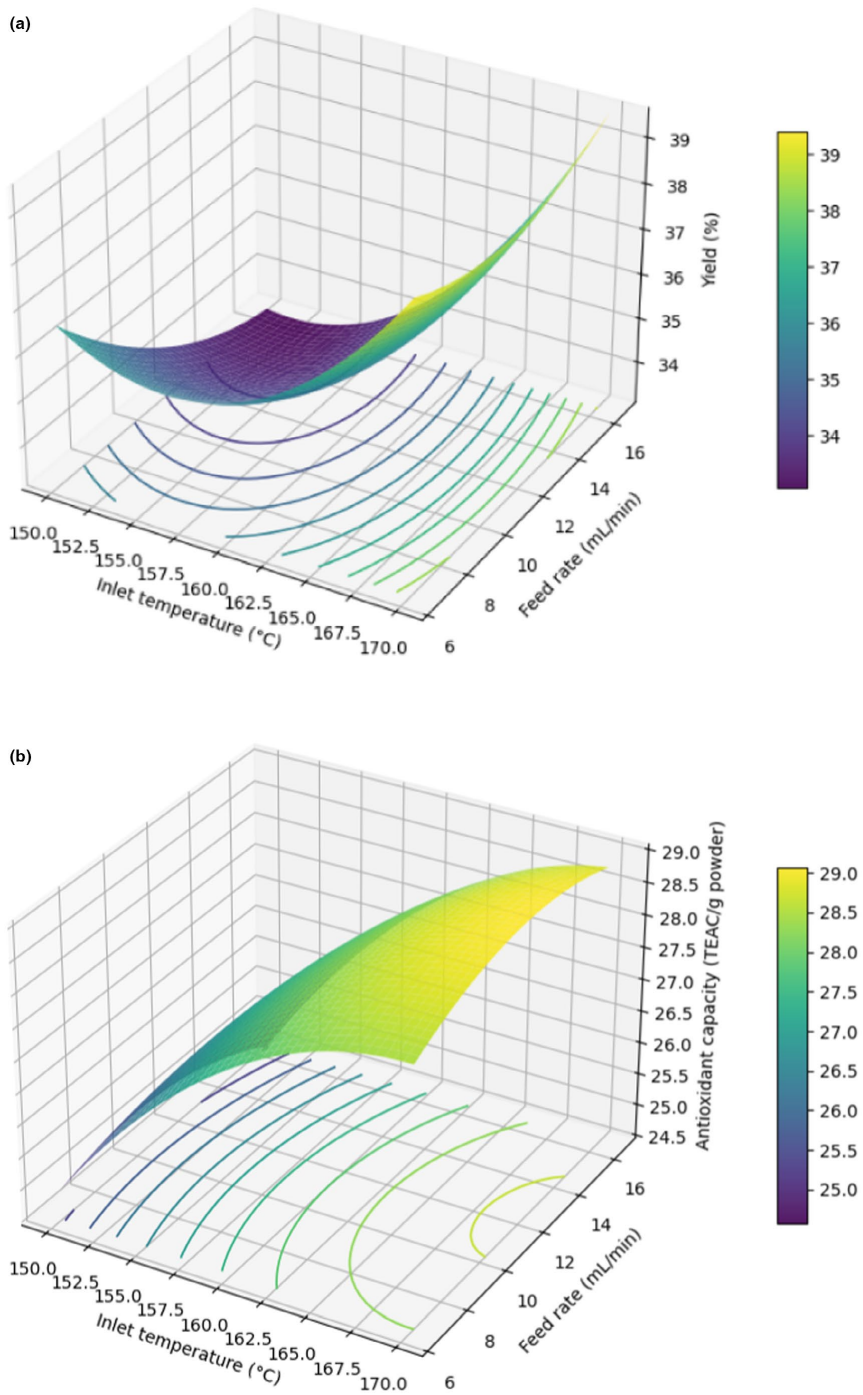
Response surface plots showing the combined effects of inlet air temperature and feed rate on (a) powder yield (%) and (b) antioxidant capacity (TEAC/g powder) of instant soluble linden tea powder produced by spray drying at a fixed maltodextrin concentration of 15% (w/w). Contour projections at the base of each surface illustrate the interaction between process variables and highlight the regions of optimal response.

The effects of spray drying parameters on the quality attributes of instant linden tea powder were evaluated using response surface methodology. The combined influence of inlet air temperature (X_1_), feed rate (X_2_), and maltodextrin concentration (X_3_) on yield, moisture content, antioxidant activity, and total phenolic content was systematically analyzed to elucidate process–structure–function relationships. The experimental design matrix and measured responses are presented in Table 1 (see also Figure S1 for a visual overview), while the three‐dimensional response surface plots illustrating the combined effects of processing variables are shown in Figure 5. ANOVA results confirmed that the developed quadratic models were statistically significant (*p* < 0.05) with a non‐significant lack‐of‐fit, indicating good agreement between experimental and predicted values. The corresponding statistical parameters are summarized in Tables S1–S4. The second‐order polynomial regression equations describing the relationships between spray drying variables and each response variable are provided in Table S5. The coefficient of determination (*R* ^2^) values for yield, moisture content, and antioxidant activity were 91.27%, 95.93%, and 95.71%, respectively, indicating that the fitted quadratic models explained most of the variability in the responses. The adjusted *R* ^2^ values (75.54%, 88.61%, and 87.98%) were in reasonable agreement with the *R* ^2^ values, confirming the adequacy of the models. The predicted *R* ^2^ values were 0.00% for yield, 38.01% for moisture content, and 33.08% for antioxidant activity. The low predicted *R* ^2^ for yield indicates limited predictive capability for new observations, which is not uncommon in small experimental designs (*n* = 15) with complex quadratic models. Nevertheless, the validation experiments performed under the optimized conditions confirmed good agreement between predicted and experimental values, supporting the practical utility of the models.

**TABLE 1:** Box–Behnken experimental design matrix and corresponding experimental responses for spray drying of linden tea infusion.

Run	X_1_: Temperature (°C)	X_2_: feed rate (mL/min)	X_3_: MD (%)	Yield (%)	Moisture (%)	Antioxidant capacity (TEAC/g)	Total phenol (GAEq)
1	170	11.5	10	41.397	3.94	35.105	118.8
2	160	11.5	15	28.571	3.54	28.813	87.40
3	170	11.5	20	45.001	2.17	19.744	54.80
4	150	16.5	15	28.473	4.21	24.050	68.10
5	150	11.5	10	33.692	5.17	34.848	91.50
6	150	6.50	15	35.547	3.97	25.396	75.20
7	160	6.50	10	34.024	4.41	32.618	97.95
8	160	16.5	10	37.230	4.67	32.907	89.65
9	150	11.5	20	40.328	4.11	15.345	57.61
10	160	6.50	20	41.708	2.77	20.077	59.65
11	170	16.5	15	33.454	3.71	30.013	83.05
12	160	11.5	15	29.494	3.42	29.774	87.95
13	160	11.5	15	31.121	3.61	28.751	88.15
14	160	16.5	20	42.477	3.81	21.346	74.70
15	170	6.50	15	36.814	2.84	30.538	72.55

#### 3.4.1 Effect of Spray Drying Parameters on Yield and Moisture Content

The spray drying yield ranged from 28% to 43%, depending on inlet air temperature, feed rate, and maltodextrin concentration (Figure 5a). Yield increased significantly with increasing inlet temperature, particularly at lower feed rates (6.5–10 mL min^−1^), indicating improved drying efficiency and reduced wall deposition. Higher inlet temperatures enhance the driving force for moisture evaporation, leading to faster crust formation around droplets and reduced particle stickiness, which collectively improve powder recovery (Mazár et al. 2025; Thi Anh Dao et al. 2021).

Increasing maltodextrin concentration positively influenced yield and moisture reduction by increasing the glass transition temperature of the solids and forming a protective matrix around droplets (Mazár et al. 2025; Figure S2). This carrier effect reduced stickiness and promoted stable particle formation, even under relatively high feed rate conditions.

At lower inlet temperatures, combined with high feed rates, the yield decreased markedly, and the moisture content increased (Figure S3). Insufficient thermal energy and shortened residence time limited effective moisture removal, leading to partially dried particles with higher surface moisture and increased adhesion to the drying chamber walls. These observations are consistent with classical spray drying theory, where the vapor pressure gradient between the droplet surface and the drying air governs the mass transfer driving force.

The response surface and contour plot (Figure 5a) illustrate the interaction effect between inlet air temperature and feed rate on powder yield. The steep slope at higher temperatures suggests that thermal energy is the dominant factor controlling moisture removal and particle formation. The elliptical shape of the contour lines indicates a significant interaction between temperature and feed rate, confirming that the combined effect of these variables on yield is greater than their individual effects.

#### 3.4.2 Antioxidant Activity and Total Phenolic Content

Antioxidant activity of spray‐dried linden tea powders varied considerably across the experimental runs, ranging from 15.3 to 35.1 mmol Trolox g^−1^ (Table 1). The response surface model indicated that antioxidant activity slightly increased with increasing inlet air temperature, while feed rate had no significant effect (*p* > 0.05). Figure 5b shows that antioxidant activity increased with increasing inlet temperature, with the slight curvature in the response surface indicating that higher temperatures promote the formation of epimerized catechins that retain or enhance radical scavenging capacity. The nearly parallel contour lines along the feed rate axis confirm that feed rate did not significantly affect antioxidant activity (*p* = 0.960), consistent with the ANOVA results. Although high‐temperature processes are often associated with polyphenol degradation, the presence of maltodextrin acted as a thermal protective agent during the short residence time of spray drying, preserving catechins and their derivatives. This protective effect is attributed to the formation of a glassy matrix around phenolic compounds during atomization and drying, which limits oxygen exposure and reduces thermal degradation (Mazár et al. 2025). The rapid moisture removal further minimizes the time available for heat‐induced reactions, allowing thermally sensitive compounds to retain their structural integrity.

Moreover, the rise in antioxidant capacity at higher drying temperatures may be attributed to the formation of epimerization products with high radical‐scavenging potential. Previous studies have shown that catechin epimerization can occur at temperatures above 145°C (Wang et al. 2008), generating compounds such as GCG with comparable or enhanced antioxidant activity. This behavior is consistent with the infusion kinetics results of the present study, where increased concentrations of (−)‐EGCG and epimerized catechins correlated with enhanced antioxidant capacity. The antioxidant values obtained in this study compare favorably with those reported for other spray‐dried herbal teas. For instance, Ayaz et al. (2026) reported that spray‐dried dandelion leaf extract encapsulated with maltodextrin exhibited TEAC values within a similar range, with optimal retention of antioxidant activity achieved at inlet temperatures around 160°C–170°C.

The response surface plot for TPC is shown in Figure S4. Total phenolic content (TPC) was initially included as a response variable in the Box–Behnken design. However, the quadratic model for TPC exhibited a highly significant lack‐of‐fit (*p* = 0.002), indicating that the model did not adequately fit the experimental data (see Table S4). Examination of the model parameters revealed that maltodextrin concentration was the only significant factor (*p* < 0.001), while inlet temperature (*p* = 0.137) and feed rate (*p* = 0.646) showed no significant effects. Although the *R* ^2^ (93.33%) and adjusted *R* ^2^ (81.33%) values were relatively high, the significant lack of fit suggests that the model is overfitted and has limited predictive capability. This is likely because the variation in total phenolic compounds was dominated by the diluting effect of maltodextrin in the powder matrix, overshadowing the more subtle effects of processing parameters. It is well established that Box–Behnken designs are primarily intended for estimating second‐order models and have limited ability to detect or estimate higher‐order terms when lack‐of‐fit occurs (Arshad et al. 2012). Similar observations have been reported in other studies employing Box–Behnken design, where significant lack‐of‐fit was encountered and the authors acknowledged that third‐order terms could not be estimated appropriately due to aliasing with linear effects (Barabadi et al. 2019; Sugar et al. 2025). Therefore, TPC results are presented in tabular form (Table 1) and discussed descriptively, rather than being included in the response surface models.

#### 3.4.3 Process Optimization and Validation

Multi‐response optimization based on desirability functions was applied to simultaneously maximize powder yield, antioxidant activity, and total phenolic content while targeting a moisture content of 4%. The optimization goals were set as follows: maximize yield (target: 45%), maximize antioxidant activity (target: 35 mmol Trolox g^−1^), maximize total phenolic content (target: 118.8 mg GAE g^−1^), and set moisture content to a target value of 4% (acceptable range: 2.17%–5.17%). The overall desirability function (D) combines these individual goals into a single composite score ranging from 0 to 1. The optimal spray drying conditions were identified as an inlet air temperature of 170°C, a feed rate of 12.28 mL min^−1^, and a maltodextrin concentration of 10.04%, yielding an overall desirability value of 0.8185.

Due to equipment limitations, the feed rate was adjusted to 10 mL min^−1^ (the nearest achievable value) and the maltodextrin concentration was set to 10% w/v. To validate the predictive capability of the response surface model, independent experiments were performed in triplicate under the optimized conditions (170°C, 10 mL min^−1^, 10% maltodextrin). Table 2 compares the predicted values from the model with the experimentally obtained results; to validate the predictive capability of the response surface model, an independent experiment was performed under the optimized conditions.

**TABLE 2:** Validation of response surface models under applied optimal conditions (170°C, 10 mL/min, 10% maltodextrin).

Response	Predicted value (at theoretical optimum)	Experimental value (mean ± SD, *n* = 3)	CV (%)
Yield (%)	36.83	40.43 ± 1.66	4.10
Moisture content (%)	4.00	3.96 ± 0.27	6.82
Antioxidant activity (mmol Trolox g^−1^)	35.33	33.80 ± 2.18	6.45
Total phenol content (mg GAE g^−1^)	111.63	107.5 ± 8.28	7.70

#### 3.4.4 Solubility and Phenolic Profile Comparison

The instant linden tea powder exhibited excellent water solubility, with an average value of 99.2% ± 2.0%, confirming its suitability for instant beverage applications.

**FIGURE 6:**
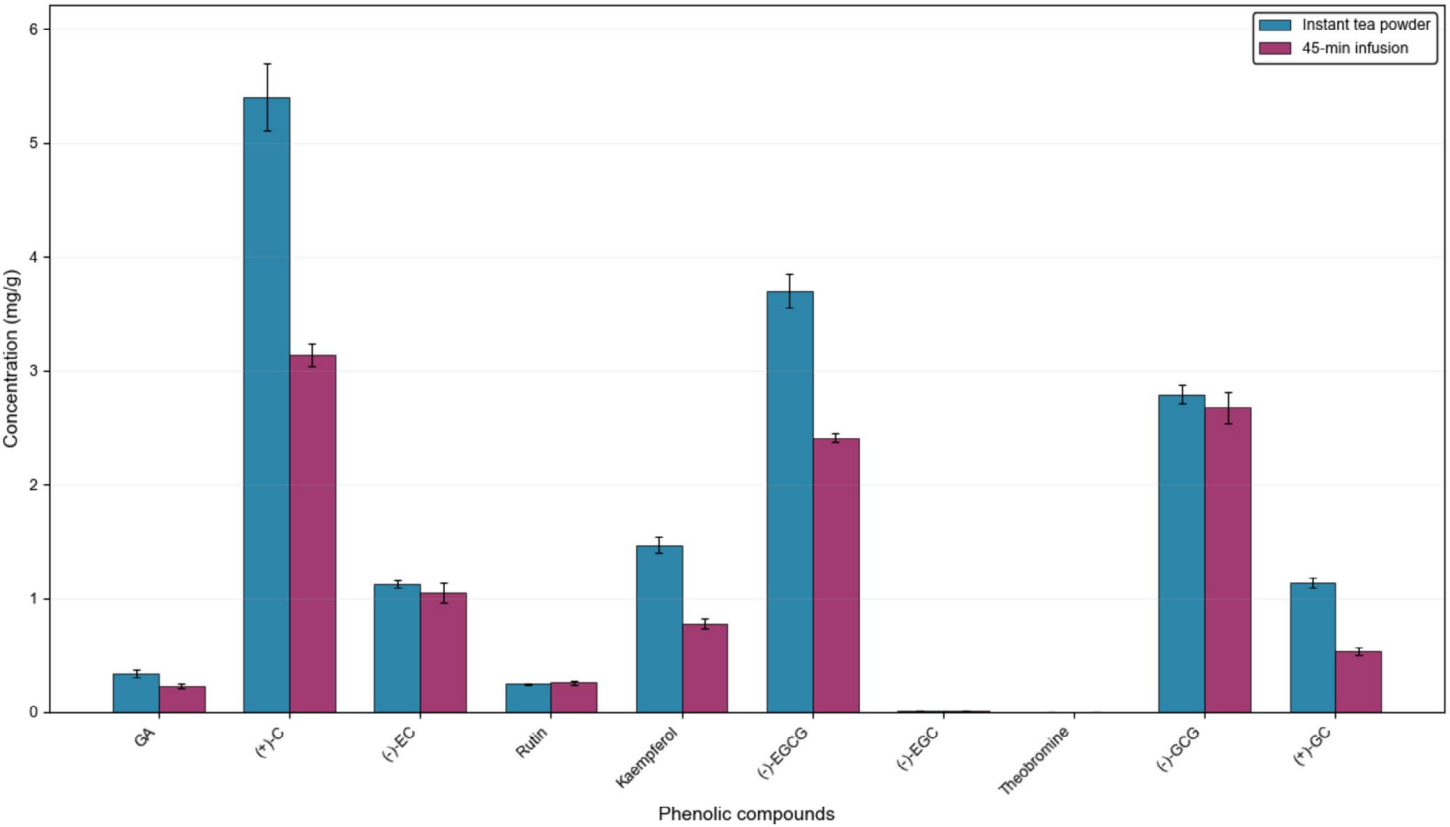
Comparison of phenolic compound concentrations between dissolved instant tea powder and traditionally prepared 45‐min linden tea infusion. Values represent means ± standard deviation (*n* = 3). C, catechin; EC, epicatechin; EGC, epigallocatechin; EGCG, epigallocatechin gallate; GA, gallic acid; GC, gallocatechin; GCG, gallocatechin gallate.

To evaluate the impact of spray drying on phenolic composition, the reconstituted powder was compared with the optimally prepared 45‐min infusion using HPLC analysis. Overlay chromatograms are provided in Figure S5, and quantitative comparisons are shown in Figure 6. The concentrations of gallic acid, (−)‐epicatechin, rutin, and (−)‐gallocatechin gallate were comparable between the instant powder and the 45‐min infusion, indicating minimal degradation or loss during drying.

Interestingly, higher concentrations of catechin, kaempferol, epigallocatechin gallate, and gallocatechin were observed in the reconstituted instant powder compared to the infusion. This may be attributed to the rapid moisture removal and short thermal exposure during spray drying, which likely limits the thermal degradation or epimerization reactions observed during extended infusion (Section 3.1). No additional or unidentified peaks were detected in the chromatograms of the instant powder, and theobromine remained at negligible levels. This is particularly relevant from a sensory perspective, as theobromine has been associated with bitterness in herbal infusions (Červenka et al. 2023). The negligible theobromine content in the instant powder therefore contributes to its favorable sensory profile, as confirmed by the sensory evaluation (Section 3.5). These findings indicate that spray drying preserved the original phenolic profile without generating detectable degradation products.

Overall, the optimized spray drying process produced an instant linden tea powder with good solubility and a phenolic composition closely matching that of the optimally prepared infusion, confirming its suitability as a convenient and stable alternative to traditional tea preparation.

### 3.5 Sensory Evaluation of Linden Tea Samples

The sensory attributes of the four linden tea samples differed significantly across most evaluated parameters (*p* < 0.05), confirming that both infusion time and reconstitution concentration influence sensory perception (Table 3).

**TABLE 3:** Mean sensory scores of linden tea samples (*n* = 10, 9‐point scale).

Attribute	Sample 523 3 min bagged	Sample 614 40 min loose	Sample 241 instant 2 g	Sample 855 instant 4 g
Appearance				
Color	6.8 ± 0.5^c^	8.0 ± 0.4^b^	8.2 ± 0.5^b^	9.4 ± 0.3^a^
Clarity	8.1 ± 0.4^b^	7.1 ± 0.5^c^	8.5 ± 0.4^ab^	9.1 ± 0.4^a^
Sediment	2.3 ± 0.4^c^	5.4 ± 0.6^a^	1.1 ± 0.3^d^	1.0 ± 0.2^d^
Aroma				
Floral	7.2 ± 0.5^b^	8.1 ± 0.5^a^	7.0 ± 0.5^b^	7.6 ± 0.5^ab^
Green/grassy	8.3 ± 0.5^a^	7.4 ± 0.5^b^	6.9 ± 0.5^c^	7.7 ± 0.5^b^
Overall	7.3 ± 0.5^c^	8.8 ± 0.4^a^	7.2 ± 0.5^c^	8.3 ± 0.5^b^
Taste				
Sweetness	6.8 ± 0.5^d^	7.5 ± 0.5^c^	8.1 ± 0.4^b^	8.9 ± 0.4^a^
Bitterness	2.4 ± 0.5^c^	5.8 ± 0.6^a^	5.2 ± 0.5^b^	5.4 ± 0.5^ab^
Floral	7.6 ± 0.5^b^	8.8 ± 0.4^a^	6.8 ± 0.5^c^	8.7 ± 0.5^a^
Mineral/herbal	7.6 ± 0.5^a^	4.2 ± 0.5^c^	5.7 ± 0.5^b^	6.4 ± 0.5^b^
Mouthfeel				
Astringency	3.1 ± 0.5^d^	6.4 ± 0.6^b^	5.1 ± 0.5^c^	7.2 ± 0.5^a^
Body	2.4 ± 0.5^d^	8.3 ± 0.5^b^	6.8 ± 0.5^c^	8.9 ± 0.4^a^
Smoothness	8.8 ± 0.4^a^	7.6 ± 0.5^b^	8.9 ± 0.4^a^	8.7 ± 0.4^a^
Warmth	8.1 ± 0.4^b^	8.5 ± 0.4^ab^	8.2 ± 0.4^b^	8.4 ± 0.4^ab^
Aftertaste				
Bitter	1.5 ± 0.4^d^	6.5 ± 0.6^a^	3.8 ± 0.5^c^	5.7 ± 0.5^b^
Astringent	2.1 ± 0.4^d^	8.8 ± 0.4^a^	6.8 ± 0.5^c^	8.3 ± 0.5^b^
Pleasant	3.5 ± 0.5^d^	7.7 ± 0.5^b^	6.5 ± 0.5^c^	8.4 ± 0.4^a^
Overall				
Acceptability	6.3 ± 0.5^d^	8.1 ± 0.4^b^	7.1 ± 0.5^c^	8.8 ± 0.4^a^

*Note:* Values are mean ± SD (*n* = 10). Different superscript letters within a row indicate significant differences (*p* < 0.05, Tukey's HSD test).

**FIGURE 7:**
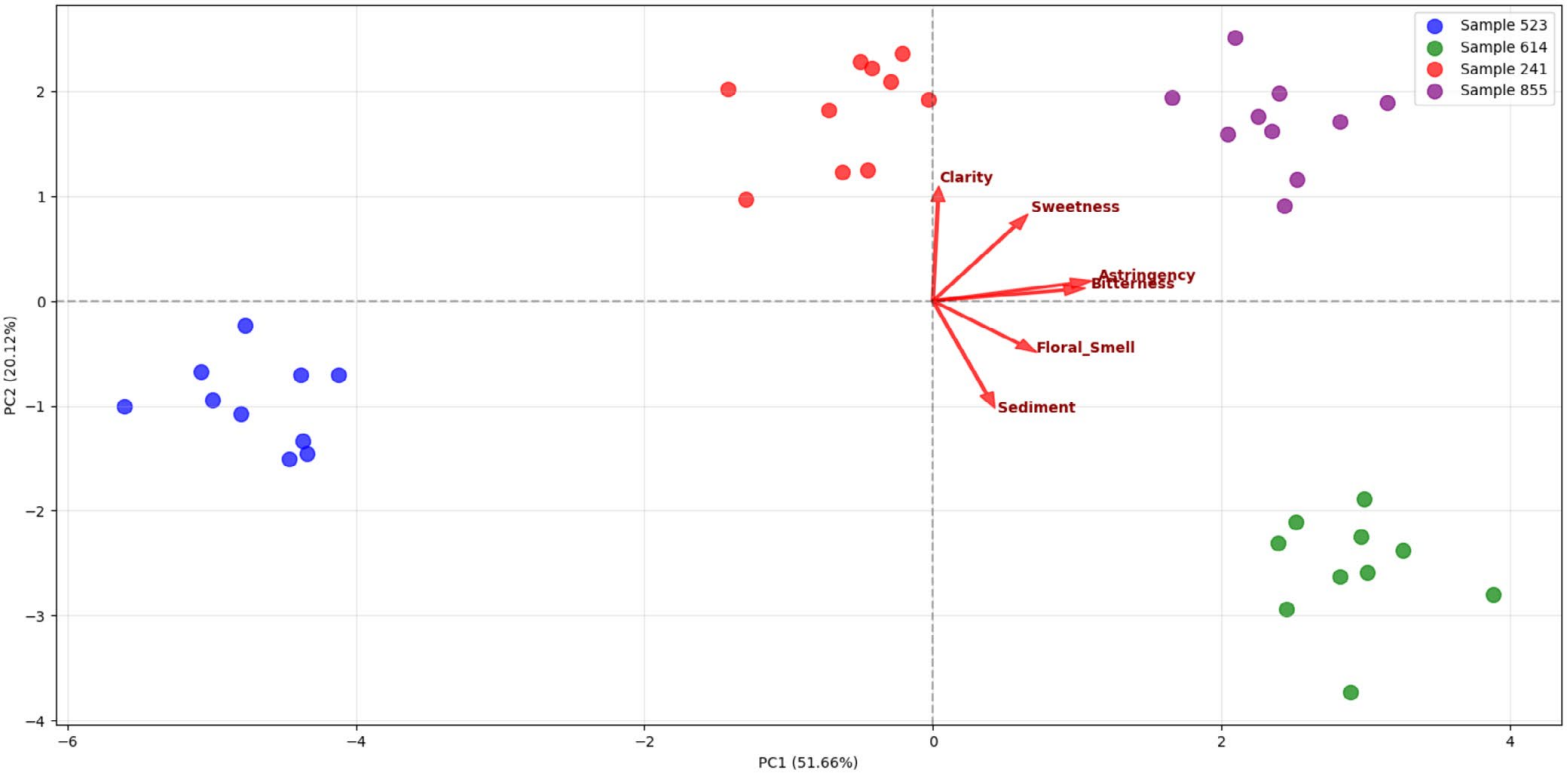
Principal component analysis biplot of sensory attributes and linden tea samples. PC1 and PC2 explain 52.3% and 26.1% of total variance, respectively.

Multivariate analysis confirmed clear differentiation among the samples (Wilks' λ = 0.000, *p* < 0.001). Principal component analysis (PCA) explained 78.4% of the total variance (Figure 7), separating samples according to infusion duration and powder concentration. The 3 min bagged tea (Sample 523) was characterized by pronounced green/grassy notes but low astringency and body. In contrast, the 40 min loose leaf tea (Sample 614) exhibited high floral characteristics with balanced astringency. The instant powder samples were separated by concentration: Sample 855 (4 g/50 mL) showed high color intensity, sweetness, body, and astringency, while Sample 241 (2 g/50 mL) displayed intermediate sensory profiles.

The 40‐min infusion (Sample 614) received significantly higher scores than the 3‐min commercial tea bag infusion (Sample 523) for color, floral aroma, floral taste, and overall aroma (*p* < 0.05). This improvement is consistent with the enhanced extraction of phenolic compounds and flavonoids during infusion optimization (Section 3.1). Astringency and bitterness also increased with infusion time, reflecting greater extraction of catechins and related phenolic compounds. Despite these increases, overall acceptability improved significantly, suggesting that the characteristic bitterness and astringency of properly brewed linden tea contributed positively to sensory perception.

Among instant samples, the higher reconstitution level (Sample 855, 4 g/50 mL) produced significantly higher scores for color, sweetness, body, and overall acceptability compared to the lower concentration (Sample 241, 2 g/50 mL). The increased body and perceived sweetness are attributable to the higher solids content and maltodextrin concentration, while elevated astringency reflects increased levels of phenolic compounds. Notably, the floral taste of Sample 855 was comparable to that of the 40‐min loose leaf infusion (Sample 614), indicating that spray drying effectively preserved the characteristic flavor compounds of linden tea.

Correlation analysis revealed strong relationships between sensory perception and chemical composition (Table 4). Astringency correlated strongly with epigallocatechin gallate (EGCG; *r* = 0.89, *p* < 0.01) and gallocatechin gallate (GCG; *r* = 0.84, *p* < 0.05). Bitterness showed the strongest association with theobromine (*r* = 0.92, *p* < 0.01). Sweetness was most strongly correlated with maltodextrin content (*r* = 0.94, *p* < 0.01), confirming that perceived sweetness derives primarily from the carrier. Body correlated strongly with both maltodextrin (*r* = 0.88) and rutin (*r* = 0.79). The strong correlations observed between specific sensory attributes and chemical compounds identify the key drivers of liking for instant linden tea. Maltodextrin emerged as the primary driver of sweetness (*r* = 0.94) and body (*r* = 0.88), while catechin content was the main contributor to floral taste (*r* = 0.84) and overall acceptability (*r* = 0.82). Overall acceptability showed the strongest correlations with maltodextrin (*r* = 0.91) and catechin (*r* = 0.82), suggesting that consumer preference is driven by a balance between sweetness, body, and authentic linden flavor.

**TABLE 4:** Pearson correlation coefficients between sensory attributes and bioactive compounds.

Sensory attribute	EGCG	GCG	Catechin	Theobromine	Maltodextrin
Astringency	0.89**	0.84*	−0.45	0.71	0.58
Bitterness	0.81*	0.76	−0.52	0.92**	0.43
Sweetness	−0.38	−0.42	0.65	−0.58	0.94**
Body	0.72	0.68	0.71	0.64	0.88**
Floral taste	0.45	0.38	0.84*	−0.62	0.51
Overall acceptability	0.69	0.65	0.82*	0.48	0.91**

* *p* < 0.05.

** *p* < 0.01.

These sensory results validate the process optimization decisions. The 40‐min infusion, previously identified as optimal based on extraction kinetics, antioxidant activity, and color development, received significantly higher overall acceptability (8.1) than the 3‐min bagged tea (6.3). Among the instant samples, the higher reconstitution level (4 g/50 mL) was strongly preferred (8.8 vs. 7.1), indicating that this concentration provides a more favorable sensory profile. The sensory performance of Sample 855 confirms that spray drying with 10% maltodextrin effectively preserved the characteristic linden tea attributes while offering the convenience of an instant format.

## 4 Conclusion

This study demonstrates that integrating infusion kinetics with response surface‐optimized spray drying enables the production of instant linden tea with controlled bioactive composition and high process efficiency. Kinetic analysis identified infusion at 80°C for 45 min as the optimal extraction condition, maximizing flavonoid recovery while limiting undesirable thermal transformations. Spray drying of the optimized infusion, using maltodextrin as a carrier, produced a stable and highly soluble powder that preserved the phenolic profile and antioxidant functionality of the freshly prepared infusion. The compound level approach adopted in this study, tracking individual flavonoids rather than relying solely on bulk phenolic measurements, provided critical insights into the behavior of specific bioactive compounds during both extraction and drying. This level of detail is essential for designing processes that preserve not only the quantity but also the functional quality of phenolic compounds in instant tea products.

Sensory evaluation confirmed that both the optimized infusion and the reconstituted instant powder were well accepted, with the higher reconstitution level yielding the highest overall acceptability. Correlation analysis further indicated that sensory preference was associated with the balance between carrier‐induced mouthfeel and characteristic linden phenolic compounds. Future studies could further investigate the bioaccessibility of these compounds during simulated gastrointestinal digestion, as well as the long‐term stability of the instant powder under different storage conditions. Pilot‐scale validation and application of the same integrated approach to other herbal teas are also recommended for future research.

Overall, these findings provide a scalable and reproducible processing strategy for producing instant linden tea powders with standardized bioactive composition, functional stability, and consumer‐acceptable sensory quality. This integrated approach offers a practical framework for industrial production of instant herbal teas with consistent composition and improved convenience.

## Author Contributions

**Evren Altiok:** conceptualization, methodology, validation, investigation, supervision, writing – review and editing, writing – original draft, resources, visualization, funding acquisition, project administration, software. **Ceyhun Kasapoglu:** formal analysis, visualization, investigation, methodology, data curation, writing – review and editing.

## Funding

The authors have nothing to report.

## Ethics Statement

Ethical review and approval were waived for this study because the sensory evaluation involved only the assessment of food products by healthy adult volunteers, did not include any invasive procedures, and did not collect sensitive personal data. All participants were informed about the purpose of the study and provided voluntary informed consent prior to participation. The study was conducted in accordance with the ethical standards of the institutional research committee.

## Conflicts of Interest

The authors declare no conflicts of interest.

## Supporting information


**Figure S1:** Comparison of yield, moisture, antioxidant activity (TEAC/g), and total phenolic content (mg GAE/g) responses across all experimental runs (Run 1–15) of the Box–Behnken design. Values represent mean ± SD (*n* = 3). This figure provides a visual overview of the variability in each response and allows rapid identification of runs with maximum and minimum values for each quality attribute.
**Figure S2:** Effect of maltodextrin concentration on (a) powder yield and (b) antioxidant activity at fixed inlet temperature (160°C) and feed rate (11.5 mL/min). Curves represent model predictions obtained from second‐order polynomial regression. The non‐linear behavior of yield reflects the role of maltodextrin in modifying particle stickiness and glass transition properties during drying.
**Figure S3:** Response surface and contour plots showing the combined effects of inlet temperature and feed rate on powder moisture content at 15% maltodextrin concentration.
**Figure S4:** Response surface plot illustrating the effect of inlet temperature and feed rate on total phenolic content of spray‐dried linden tea powder at 15% maltodextrin concentration.
**Figure S5:** Overlay HPLC chromatograms showing the catechin and flavonoid profiles of linden tea obtained by conventional infusion (blue) and instant linden tea prepared by dissolving the spray‐dried powder in water (red). The chromatograms indicate comparable qualitative profiles with no additional or undesired peaks detected in the instant tea sample.
**Table S1:** Analysis of variance (ANOVA) for the quadratic response surface model fitted to powder yield (%) during spray drying of linden tea extract.
**Table S2:** Analysis of variance (ANOVA) for the quadratic response surface model fitted to moisture content (%) of spray‐dried linden tea powder.
**Table S3:** Analysis of variance (ANOVA) for the quadratic response surface model fitted to antioxidant activity (TEAC g^−1^) of spray‐dried linden tea powder.
**Table S4:** Analysis of variance (ANOVA) for the quadratic response surface model fitted to total phenolic content (mg GAE g^−1^) of spray‐dried linden tea powder.
**Table S5:** Second‐order polynomial equations describing the relationships between spray drying variables and the selected responses.
**Table S6:** Response factors (RF) (μgmL^−1^)/(mAU·s) (μg mL^−1^)/(mAU·s) (μgmL^−1^)/(mAU·s) and relative response factors (RRF) of catechin standards determined in this study. Expressed relative to (−)‐EGCG. (−)‐EC. and (+)‐catechin.
**Table S7:** Response factors (RF) and relative response factors (RRF) of catechin standards reported in the literature [26]. Expressed relative to (−)‐EGCG. (−)‐EC. and (+)‐catechin.

## Data Availability

The data that support the findings of this study are available from the corresponding author upon reasonable request.
