# Supplementary material for: Production of Instant Linden Tea (Tilia spp.) Powder: Integrating Compound‐Level Infusion Kinetics With Response Surface‐Optimized Spray Drying
Source: Food Sci Nutr. 2026 Jun 12;14(6):e72018. doi: 10.1002/fsn3.72018 (PMC13263237; doi:10.1002/fsn3.72018)
Supplement: Supplementary file 1 — Figure S1: Comparison of yield, moisture, antioxidant activity (TEAC/g), and total phenolic content (mg GAE/g) responses across all experimental runs (Run 1–15) of the Box–Behnken design. Values represent mean ± SD (n = 3). This figure provides a visual overview of the variability in each response and allows rapid identification of runs with maximum and minimum values for each quality attribute. Figure S2: Effect of maltodextrin concentration on (a) powder yield and (b) antioxidant activity at fixed inlet temperature (160°C) and feed rate (11.5 mL/min). Curves represent model predictions obtained from second‐order polynomial regression. The non‐linear behavior of yield reflects the role of maltodextrin in modifying particle stickiness and glass transition properties during drying. Figure S3: Response surface and contour plots showing the combined effects of inlet temperature and feed rate on powder moisture content at 15% maltodextrin concentration. Figure S4: Response surface plot illustrating the effect of inlet temperature and feed rate on total phenolic content of spray‐dried linden tea powder at 15% maltodextrin concentration. Figure S5: Overlay HPLC chromatograms showing the catechin and flavonoid profiles of linden tea obtained by conventional infusion (blue) and instant linden tea prepared by dissolving the spray‐dried powder in water (red). The chromatograms indicate comparable qualitative profiles with no additional or undesired peaks detected in the instant tea sample. Table S1: Analysis of variance (ANOVA) for the quadratic response surface model fitted to powder yield (%) during spray drying of linden tea extract. Table S2: Analysis of variance (ANOVA) for the quadratic response surface model fitted to moisture content (%) of spray‐dried linden tea powder. Table S3: Analysis of variance (ANOVA) for the quadratic response surface model fitted to antioxidant activity (TEAC g−1) of spray‐dried linden tea powder. Table S4: Analysis of variance (ANOVA) for t [file FSN3-14-e72018-s001.docx]

**Supplementary Material**

The following supporting information accompanies this article:

Supplementary Index

Item Description Section

Table S1 ANOVA for powder yield S1.2

Table S2 ANOVA for moisture content S1.2

Table S3 ANOVA for antioxidant activity S1.2

Table S4 ANOVA for total phenolic content S1.2

Table S5 Second-order polynomial regression equation S1.3

Table S6 Response factors (RF) and relative response factors (RRF) of S2.2 catechin standard (this study)

Table S7 Response factors (RF) and relative response factors (RRF) of S2.2 catechin standards (literature)

Figure S1 Overview of experimental responses (yiled, moisture, S1.1 antioxidant activity, TPC)

Figure S2 Effect of maltodextrin concentration on powder yield and S1.4 antioxidant activity

Figure S3 Response surface plot for moisture content S1.5

Figure S4 Response surface plot for total phenolic content S1.6

Figure S5 Overlay HPLC chromatograms (infusion vs. reconstituted S2.1 powder)

**S1 – Experimental design and model statistics**

**S1.1. Overview of Experimental Responses**

According to Box–Behnken experimental design matrix, corresponding response values for spray drying of linden tea infusion are presented in Figure 1.


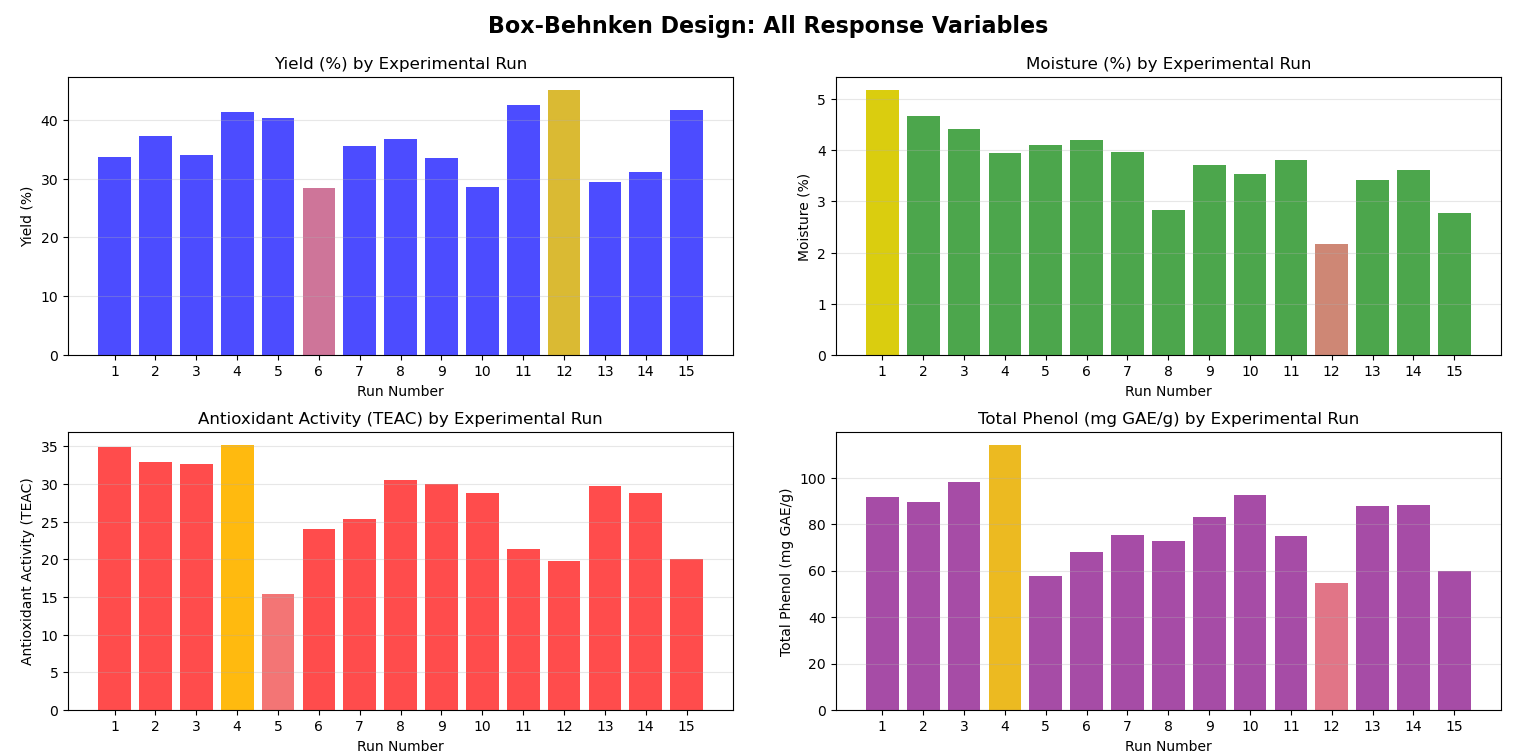


**Figure S1.** Comparison of yield, moisture, antioxidant activity (TEAC/g), and total phenolic content (mg GAE/g) responses across all experimental runs (Run 1-15) of the Box–Behnken design. Values represent mean ± SD (n=3). This figure provides a visual overview of the variability in each response and allows rapid identification of runs with maximum and minimum values for each quality attribute.

**S1.2. Statistical analysis: ANOVA Tables for response variables**

**Table S1.** Analysis of variance (ANOVA) for the quadratic response surface model fitted to powder yield (%) during spray drying of linden tea extract.

| Source | | | | | | | DF | Adj SS | Adj MS | F-Value | p-Value |
| --- | --- | --- | --- | --- | --- | --- | --- | --- | --- | --- | --- |
| Model | | | | | | | 9 | 364.564 | 40.507 | 5.81 | 0.034 |
| Linear | | | | | | | 3 | 115.693 | 3.564 | 5.53 | 0.048 |
| Temperature (C) | | | | | | | 1 | 43.366 | 43.366 | 6.21 | 0.055 |
| Feed rate (mL/min) | | | | | | | 1 | 5.215 | 5.215 | 0.75 | 0.427 |
| MD (%) | | | | | | | 1 | 67.112 | 64.112 | 9.62 | 0.027 |
| Square | | | | | | | 3 | 241.640 | 80.547 | 11.54 | 0.011 |
| Temperature(C)* Temperature(C) | | | | | | | 1 | 23.897 | 23.897 | 3.42 | 0.123 |
| Feed rate (mL/min)* Feed rate (mL/min) | | | | | | | 1 | 6.233 | 6.233 | 0.89 | 0.388 |
| MD (%)*MD (%) | | | | | | | 1 | 226.475 | 226.475 | 32.46 | 0.002 |
| 2-Way Interaction | | | | | | | 3 | 7.231 | 2.410 | 0.35 | 0.795 |
| Temperature(C) *Feed rate (mL/min) | | | | | | | 1 | 3.448 | 3.448 | 0.49 | 0.513 |
| Temperature(C) *MD (%) | | | | | | | 1 | 2.298 | 2.298 | 0.33 | 0.591 |
| Feed rate (mL/min)*MD (%) | | | | | | | 1 | 1.485 | 1.485 | 0.21 | 0.664 |
| Error | | | | | | | 5 | 34.889 | 6.978 |  |  |
| Lack-of-Fit | | | | | | | 3 | 31.555 | 10.518 | 6.31 | 0.140 |
| Pure error | | | | | | | 2 | 3.334 | 1.667 |  |  |
| Total | | | | | | | 14 | 399.453 |  |  |  |
| Model Summary | | | | | | |  |  |  |  |  |
| S | R-sq | R-sq(adj) | R-sq (pred) | | |  |  |  |  |  |  |
| 2.64154 | 91.27% | 75.54% | 0.00% |  |  |  |  |  |  |  |  |

**Note:** The quadratic model for powder yield was statistically significant (p = 0.034) with a non-significant lack-of-fit (p = 0.140), indicating adequate model fit. The model explained 91.27% of the variability (R²), with an adjusted R² of 75.54%. Maltodextrin concentration and its quadratic effect were identified as significant factors (p < 0.05), while interaction terms were not significant.

**Table S2.** Analysis of variance (ANOVA) for the quadratic response surface model fitted to moisture content (%) of spray-dried linden tea powder.

| Source | | | | | | | DF | Adj SS | Adj MS | F-Value | p-Value |
| --- | --- | --- | --- | --- | --- | --- | --- | --- | --- | --- | --- |
| Model | | | | | | | 9 | 7.85309 | 0.87257 | 13.10 | 0.006 |
| Linear | | | | | | | 3 | 7.15712 | 2.38571 | 35.82 | 0.001 |
| Temperature (C) | | | | | | | 1 | 2.88000 | 2.88000 | 43.24 | 0.001 |
| Feed rate (mL/min) | | | | | | | 1 | 0.72601 | 0.72601 | 10.90 | 0.021 |
| MD (%) | | | | | | | 1 | 3.55111 | 3.55111 | 53.31 | 0.001 |
| Square | | | | | | | 3 | 0.31862 | 0.10621 | 1.59 | 0.302 |
| Temperature(C)* Temperature(C) | | | | | | | 1 | 0.00776 | 0.00776 | 0.12 | 0.747 |
| Feed rate (mL/min)* Feed rate (mL/min) | | | | | | | 1 | 0.04743 | 0.04743 | 0.71 | 0.437 |
| MD (%)*MD (%) | | | | | | | 1 | 0.28604 | 0.28604 | 4.29 | 0.093 |
| 2-Way Interaction | | | | | | | 3 | 0.37735 | 0.12578 | 1.89 | 0.249 |
| Temperature(C) *Feed rate (mL/min) | | | | | | | 1 | 0.09923 | 0.09923 | 1.49 | 0.277 |
| Temperature(C) *MD (%) | | | | | | | 1 | 0.12602 | 0.12602 | 1.89 | 0.227 |
| Feed rate (mL/min)*MD (%) | | | | | | | 1 | 0.15210 | 0.15210 | 2.28 | 0.191 |
| Error | | | | | | | 5 | 0.33304 | 0.06661 |  |  |
| Lack-of-Fit | | | | | | | 3 | 0.31458 | 0.10486 | 11.36 | 0.082 |
| Pure error | | | | | | | 2 | 001847 | 0.00923 |  |  |
| Total | | | | | | | 14 | 8.18613 |  |  |  |
| Model Summary | | | | | | |  |  |  |  |  |
| S | R-sq | R-sq(adj) | R-sq (pred) | | |  |  |  |  |  |  |
| 0.258086 | 95.93% | 88.61% | 38.01% |  |  |  |  |  |  |  |  |

**Table S3.** Analysis of variance (ANOVA) for the quadratic response surface model fitted to antioxidant activity (TEAC g⁻¹) of spray-dried linden tea powder.

| Source | | | | | | | DF | Adj SS | Adj MS | F-Value | p-Value |
| --- | --- | --- | --- | --- | --- | --- | --- | --- | --- | --- | --- |
| Model | | | | | | | 9 | 485.989 | 53.999 | 12.38 | 0.006 |
| Linear | | | | | | | 3 | 465.687 | 155.229 | 35.60 | 0.001 |
| Temperature (C) | | | | | | | 1 | 31.051 | 31.051 | 7.12 | 0.044 |
| Feed rate (mL/min) | | | | | | | 1 | 0.012 | 0.012 | 0.00 | 0.960 |
| MD (%) | | | | | | | 1 | 434.624 | 434.624 | 99.67 | 0.000 |
| Square | | | | | | | 3 | 15.604 | 5.201 | 1.19 | 0.402 |
| Temperature(C)* Temperature(C) | | | | | | | 1 | 4.032 | 4.032 | 0.92 | 0.380 |
| Feed rate (mL/min)* Feed rate (mL/min) | | | | | | | 1 | 1.193 | 1.193 | 0.27 | 0.623 |
| MD (%)*MD (%) | | | | | | | 1 | 12.059 | 12.059 | 2.77 | 0.157 |
| 2-Way Interaction | | | | | | | 3 | 4.698 | 1.566 | 0.36 | 0.786 |
| Temperature(C) *Feed rate (mL/min) | | | | | | | 1 | 0.169 | 0.169 | 0.04 | 0.852 |
| Temperature(C) *MD (%) | | | | | | | 1 | 4.289 | 4.289 | 0.98 | 0.367 |
| Feed rate (mL/min)*MD (%) | | | | | | | 1 | 0.240 | 0.240 | 0.06 | 0.824 |
| Error | | | | | | | 5 | 21.804 | 4.361 |  |  |
| Lack-of-Fit | | | | | | | 3 | 21.146 | 7.049 | 21.43 | 0.045 |
| Pure error | | | | | | | 2 | 0.658 | 0.329 |  |  |
| Total | | | | | | | 14 | 507.793 |  |  |  |
| Model Summary | | | | | | |  |  |  |  |  |
| S | R-sq | R-sq(adj) | R-sq (pred) | | |  |  |  |  |  |  |
| 2.08825 | 95.71% | 87.98% | 33.08% |  |  |  |  |  |  |  |  |

**Note on Total Phenolic Content Modeling:**

The quadratic model for total phenolic content (TPC) exhibited a highly significant lack-of-fit (p = 0.002; see Table S4 below). indicating that the model did not adequately fit the experimental data. This lack-of-fit is primarily attributed to the dominant effect of maltodextrin concentration (p < 0.001). which diluted the phenolic compounds in the powder matrix. overshadowing the more subtle effects of inlet temperature and feed rate. As discussed in detail in the main manuscript (Section 3.4.2). Box-Behnken designs have inherent limitations in estimating higher-order terms when lack-of-fit occurs [Arshad et al.. 2012; Barabadi et al.. 2019]. Therefore. TPC results are presented descriptively in Table 1 of the main manuscript and are not included in the response surface models.

**Table S4.** Analysis of variance (ANOVA) for the quadratic response surface model fitted to total phenolic content (mg GAE g⁻¹) of spray-dried linden tea powder.

| Source | | | | | | | DF | Adj SS | Adj MS | F-Value | p-Value |
| --- | --- | --- | --- | --- | --- | --- | --- | --- | --- | --- | --- |
| Model | | | | | | | 9 | 3773.04 | 419.23 | 7.78 | 0.018 |
| Linear | | | | | | | 3 | 3037.95 | 1012.65 | 18.79 | 0.004 |
| Temperature (C) | | | | | | | 1 | 169.28 | 169.28 | 3.14 | 0.137 |
| Feed rate (mL/min) | | | | | | | 1 | 12.88 | 12.88 | 0.24 | 0.646 |
| MD (%) | | | | | | | 1 | 2855.79 | 2855.79 | 52.98 | 0.001 |
| Square | | | | | | | 3 | 294.85 | 98.28 | 1.82 | 0.260 |
| Temperature(C)* Temperature(C) | | | | | | | 1 | 154.11 | 154.11 | 2.86 | 0.152 |
| Feed rate (mL/min)* Feed rate (mL/min) | | | | | | | 1 | 163.18 | 163.18 | 3.03 | 0.142 |
| MD (%)*MD (%) | | | | | | | 1 | 1.80 | 1.80 | 0.03 | 0.862 |
| 2-Way Interaction | | | | | | | 3 | 440.25 | 146.75 | 2.72 | 0.154 |
| Temperature(C) *Feed rate (mL/min) | | | | | | | 1 | 77.44 | 77.44 | 1.44 | 0.284 |
| Temperature(C) *MD (%) | | | | | | | 1 | 226.50 | 226.50 | 4.20 | 0.096 |
| Feed rate (mL/min)*MD (%) | | | | | | | 1 | 136.31 | 136.31 | 2.53 | 0.173 |
| Error | | | | | | | 5 | 269.51 | 53.90 |  |  |
| Lack-of-Fit | | | | | | | 3 | 269.21 | 89.74 | 594.94 | 0.002 |
| Pure error | | | | | | | 2 | 0.30 | 0.15 |  |  |
| Total | | | | | | | 14 | 4042.55 |  |  |  |
| Model Summary | | | | | | |  |  |  |  |  |
| S | R-sq | R-sq(adj) | R-sq (pred) | | |  |  |  |  |  |  |
| 7.34183 | 93.33% | 81.33% | 0.00% |  |  |  |  |  |  |  |  |

**S1.3. Regression equations for response variables**

The second-order polynomial model used for response surface analysis was:

Y=β_0_​+β_1_​X_1_​+β_2_​X_2_​+β_3_​X_3_​+β_12_​X_1_​X_2_​+β_13_​X_1_​X_3_​+β_23_​X_2_​X_3_​+β_11_​X_1_^2^​+β_22_​X_2_^2^​+β_33_​X_3_^2^​

where Y is the predicted response. X₁ is inlet air temperature (°C). X₂ is feed rate (mL/min). and X₃ is maltodextrin concentration (%).

**Table S5.** Second-order polynomial equations describing the relationships between spray drying variables and the selected responses.

| **Yield (%)**  Yield (%) = 708 – 7.89*T* – 3.96*F* – 6.11*M* +0.0254*T^2^*+ 0.0520*F^2^*+ 0.3133*M^2^* +0.0186*TF* – 0.0152*TM* – 0.0244*FM* |
| --- |
| **Moisture (%)**  Moisture (%) = 27.9 – 0.190*T* – 0.665*F* + 0.011*M* +0.000464*T^2^*+ 0.00453*F^2^* +0.01113*M^2^* +0.00315*TF* –0.00355*TM +* 0.00780*FM* |
| **Antioxidant activity (TEAC g^-1^)**  Antioxidant = –208 + 3.18*T* – 0.29*F* – 2.73*M* –0.0104*T^2^* – 0.0227*F^2^* – 0.0723*M^2^* +0.0041*TF* + 0.0207*TM +* 0.0098*FM* |
| **Total phenolic content (GAE g^-1^)**  Total phenol = –35.7 + 0.448*T* – 0.224*F* + 0.369*M* –0.001292*T^2^* – 0.00532*F^2^* – 0.00056*M^2^* +0.00176*TF* – 0.0030*TM +* 0.00467*FM* |

* where T is inlet air temperature (°C ). F is feed rate (mL/min). and M is maltodextrin concentration (%).

**S1.4. Effect of Maltodextrin Concentration**


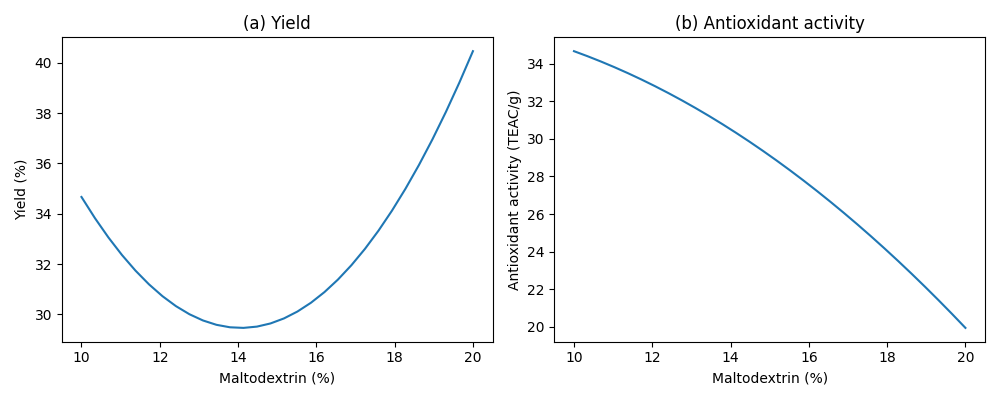


**Figure S2.** Effect of maltodextrin concentration on (a) powder yield and (b) antioxidant activity at fixed inlet temperature (160 °C) and feed rate (11.5 mL/min). Curves represent model predictions obtained from second-order polynomial regression. The non-linear behavior of yield reflects the role of maltodextrin in modifying particle stickiness and glass transition properties during drying.

**Results:**

At low maltodextrin concentrations. the low glass transition temperature of the solids resulted in increased particle stickiness and reduced powder recovery. At intermediate maltodextrin levels. atomization and drying efficiency improved; however. partial stickiness effects were still evident. Increasing maltodextrin concentration further elevated the glass transition temperature and promoted rapid surface crust formation. thereby reducing wall deposition and enhancing powder yield.

**S1.5. Moisture Content Response Surfaces**


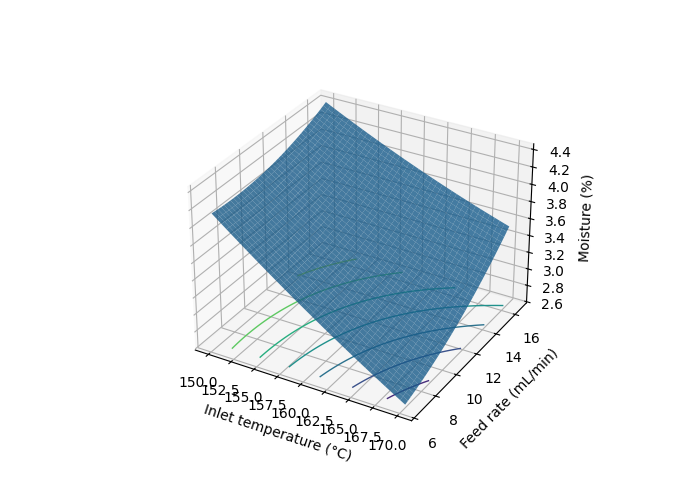


**Figure S3.** Response surface and contour plots showing the combined effects of inlet temperature and feed rate on powder moisture content at 15% maltodextrin concentration.

**S1.6. Total Phenolic Content Response Surface**


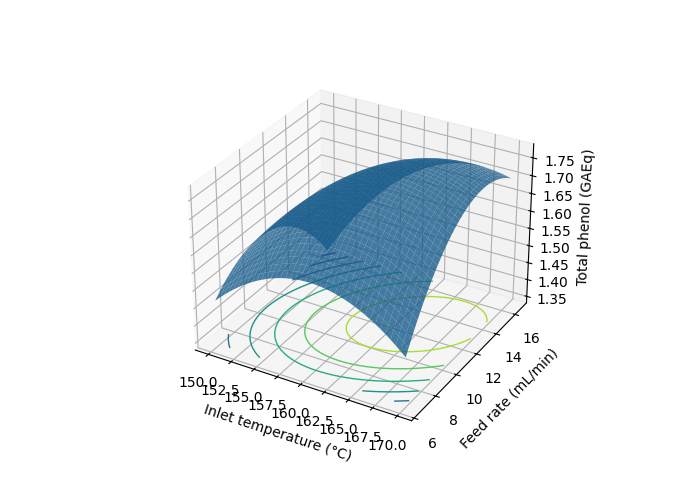


**Figure S4.** Response surface plot illustrating the effect of inlet temperature and feed rate on total phenolic content of spray-dried linden tea powder at 15% maltodextrin concentration.

**S2 HPLC Analysis**

**S2.1. HPLC chromatograms and profile comparison**

**Figure S5.** Overlay HPLC chromatograms showing the catechin and flavonoid profiles of linden tea obtained by conventional infusion (blue) and instant linden tea prepared by dissolving the spray-dried powder in water (red). The chromatograms indicate comparable qualitative profiles with no additional or undesired peaks detected in the instant tea sample.

**S2.2. Response factors and relative response factors of catechin standards**

**Table S6.** Response factors (RF) (µgmL^−1^)/(mAU⋅s)(µg mL⁻¹)/(mAU·s)(µgmL^−1^)/(mAU⋅s) and relative response factors (RRF) of catechin standards determined in this study. expressed relative to (−)-EGCG. (−)-EC. and (+)-catechin.

| Compound | RF | RRF/(-)-EGCG | RRF/ (-)-EC | RRF/ (+)-C |
| --- | --- | --- | --- | --- |
| Gallic acid | 0.02078 | 0.21043 | 0.21756 | 0.19380 |
| (+)-C | 0.10722 | 1.08577 | 1.12260 | 1.00000 |
| (-)-EGC | 0.11332 | 1.14754 | 1.18647 | 1.05689 |
| (-)-EGCG | 0.09875 | 1.00000 | 1.03392 | 0.92100 |
| (-)-EC | 0.09551 | 0.96718 | 1.00000 | 0.89078 |
| (-)-ECG | 0.10381 | 1.05124 | 1.08690 | 0.96819 |

**Table S7.** Response factors (RF) and relative response factors (RRF) of catechin standards reported in the literature [26]. expressed relative to (−)-EGCG. (−)-EC. and (+)-catechin.

| Compound | RF | RRF/(-)-EGCG | RRF/ (-)-EC | RRF/ (+)-C |
| --- | --- | --- | --- | --- |
| (+)-GC | 0.01015 | 0.89566 | 1.10926 | 1.02870 |
| (-)-EGC | 0.00984 | 0.86873 | 1.07608 | 0.99780 |
| (+)-C | 0.00986 | 0.87063 | 1.07840 | 1.00000 |
| (-)-EGCG | 0.01133 | 1.00000 | 1.23854 | 1.14850 |
| (-)-EC | 0.00914 | 0.80736 | 1.00000 | 0.92730 |
| (-)-GCG | 0.00948 | 0.83696 | 1.03659 | 0.96126 |
| (-)-ECG | 0.01031 | 0.91058 | 1.12778 | 1.04581 |
| (-)-CG | 0.00938 | 0.82836 | 1.02606 | 0.95147 |
